# A Fuzzy Hybrid MCDM Approach for Assessing the Emergency Department Performance during the COVID-19 Outbreak

**DOI:** 10.3390/ijerph20054591

**Published:** 2023-03-05

**Authors:** Miguel Ortíz-Barrios, Natalia Jaramillo-Rueda, Muhammet Gul, Melih Yucesan, Genett Jiménez-Delgado, Juan-José Alfaro-Saíz

**Affiliations:** 1 Department of Productivity and Innovation, Universidad de la Costa CUC, Barranquilla 081001, Colombia; 2 School of Transportation and Logistics, Istanbul University, Istanbul 34320, Turkey; 3 Department of Emergency Aid and Disaster Management, Munzur University, Tunceli 62000, Turkey; 4 Department of Industrial Engineering, Institución Universitaria de Barranquilla IUB, Barranquilla 080002, Colombia; 5 Research Centre on Production Management and Engineering, Universitat Politècnica de València, 46022 Valencia, Spain

**Keywords:** emergency departments (EDs), intuitionistic fuzzy analytic hierarchy process (IF-AHP), intuitionistic fuzzy decision making trial and evaluation laboratory (IF-DEMATEL), combined compromise solution (CoCoSo), performance evaluation

## Abstract

The use of emergency departments (EDs) has increased during the COVID-19 outbreak, thereby evidencing the key role of these units in the overall response of healthcare systems to the current pandemic scenario. Nevertheless, several disruptions have emerged in the practical scenario including low throughput, overcrowding, and extended waiting times. Therefore, there is a need to develop strategies for upgrading the response of these units against the current pandemic. Given the above, this paper presents a hybrid fuzzy multicriteria decision-making model (MCDM) to evaluate the performance of EDs and create focused improvement interventions. First, the intuitionistic fuzzy analytic hierarchy process (IF-AHP) technique is used to estimate the relative priorities of criteria and sub-criteria considering uncertainty. Then, the intuitionistic fuzzy decision making trial and evaluation laboratory (IF-DEMATEL) is employed to calculate the interdependence and feedback between criteria and sub-criteria under uncertainty, Finally, the combined compromise solution (CoCoSo) is implemented to rank the EDs and detect their weaknesses to device suitable improvement plans. The aforementioned methodology was validated in three emergency centers in Turkey. The results revealed that the most important criterion in ED performance was *ER facilities* (14.4%), while *Procedures and protocols* evidenced the highest positive D + R value (18.239) among the dispatchers and is therefore deemed as the main generator within the performance network.

## 1. Introduction

EDs have essential services within the scope of public health. They provide 24-h emergency health care regardless of the economic competencies of the patients [1].

The ED system has common standards for human resources, medical devices, and procedures [2]. The patient flow is not always the same but follows the following order: triage, enrollment, clinical evaluation, treatment, and discharge [2,3]. The services provided by EDs within the scope of public health in ordinary and disaster times are of vital importance. Disasters have devastating effects on health, social, and economic dimensions. Especially developing countries are affected by disasters due to insufficient resources, infrastructure that cannot respond to disasters, and the absence of disaster preparedness systems [4]. There is an imbalance between the need for health services and the provision of health services during disaster periods. In addition, health facilities inevitably become unusable due to the disaster as medical equipment is damaged, logistics instruments cannot be used, and health personnel who serve in health services become unworkable, disrupting health services [5].

COVID-19, which first appeared in December 2019, affected a particular geography and caused devastating effects on the whole world (URL-1). COVID-19 stands out from other local and other high-profile disasters as its future course is difficult to predict [6]. In most countries, EDs have been the first resort to meet patients’ complaints with various symptoms of the disease. It has proven its essential role in public health, as they evaluated the symptoms and provided isolation when deemed necessary, thereby reducing the spread of infection in the community [7]. To respond to this unusual demand, especially to deal with patients with suspected acute respiratory failure, the need to reorganize EDs has arisen [8]. The performance indicators of EDs also need to be re-evaluated due to needs such as the differentiation of demand, the increase in the need, the fact that COVID-19 generally affects the respiratory tract, the necessity of combating the risk of infection while providing healthcare services, and the protection of uninfected patients during diagnosis.

**Figure 1:**
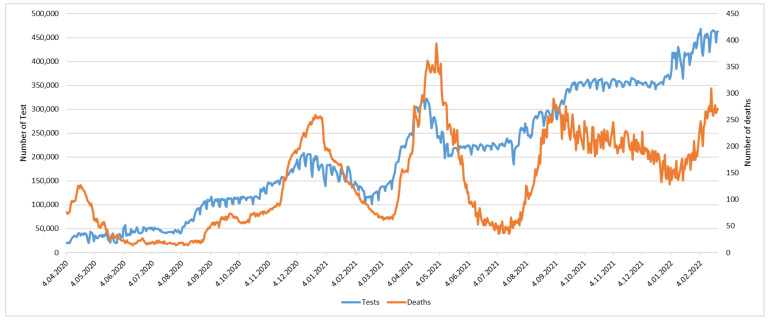
Number of tests and deaths per day.

The statistical data announced by the Turkish Ministry of Health between 4 April 2020–22 February 2022 are examined in Figure 1, where it is seen that the tests performed to identify COVID-19-positive people have increased in a short time since the beginning of the pandemic. The capacity to perform approximately 460,000 tests per day has been reached. In Turkey, the tests are carried out by the ED, which increases the workload and intensity of the ED considerably. Mortality rates appear in a waveform and have shown a downward trend recently. EDs play a key role in the fight against the pandemic. ED management needs to establish different strategies to respond to an unusual demand. Multi-criteria decision-making (MCDM) is one of the methods used in determining strategy and creating a decision support system [9]. MCDM offers two perspectives, managerial and engineering. The management perspective defines objectives and identifies and evaluates alternatives [10]. Measuring performance in sectors such as health services is challenging because there is a fine line between providing quality and effective service and efficient use of resources [11]. To determine these strategies and measure the performance of EDs, a model using an integrated intuitionistic fuzzy analytic hierarchy process (IF-AHP), intuitionistic fuzzy decision making trial and evaluation laboratory (IF-DEMATEL), and combined compromise solution (CoCoSo) approaches is proposed in this study.

The model we propose was applied in three competing hospitals in eastern Turkey, which are close enough to be used as alternatives to each other. This model will guide decision-makers to evaluate the threats and opportunities of their hospitals. The fact that the hospitals are in the same region will also show the region’s position in the fight against the outbreak.

When the literature, which will be discussed in-depth in the following section, is examined, it is seen that many researchers and health-related decision-making institutions are working on preparing units such as hospitals and emergency services in the hospital in case of epidemic diseases such as COVID-19. In some of these, the criteria used to evaluate preparedness were determined and MCDM methods were used to determine their importance. However, in the current study, the use of IF-AHP and IF-DEMATEL was preferred, unlike their counterparts. The use of the AHP-DEMATEL pair in intuitionistic fuzzy set integration is due to its ability to reflect the uncertainty in the decision-makers’ evaluations in determining the criterion weights and then revealing the relationships between them. This is because with intuitionistic fuzzy numbers, decision-makers’ evaluations are reflected in both membership and non-membership degrees. This situation strengthens the sensitivity of reflecting the uncertainty compared to classical sets. Similarly, using the CoCoSo method as the third pillar of the integrated approach is suitable because the criteria for evaluating the emergency services’ pandemic preparedness are numerical data. In this case, such an integrated approach is considered problem-specific. In particular, the contributions and advantages of the model to those in the literature are as follows:

- First, AHP, a pairwise comparison technique, was combined with intuitionistic fuzzy set theory, the advanced version of fuzzy set theory, to determine the relative importance levels of the criteria used to evaluate the COVID-19 performance of EDs. This is because AHP’s key features (hierarchical structure, pairwise comparison, consistency, etc.) include the degree of non-membership.
- While conventional methods consider the criteria independent, IF-DEMATEL considers the cause-and-effect relationships between the criteria, which is more suitable for evaluating the criteria in EDs. With IF-DEMATEL, it also provided the opportunity to present cause–effect relationships and critical ones to decision-makers in a visual structure. Thus, it provides a framework for decision-makers about how future improvements can affect the whole system.
- Finally, the CoCoSo method is adopted to determine the performance of emergency services in Turkey. Since this method is a combination of simple additive weighting (SAW), weighted aggregated product evaluation (WASPAS), and multiplicative exponential weighting (MEW) methods, it is stated in the literature that it gives more reliable results than these three methods [12]. As a result of CoCoSo being a holistic method, it allows a more robust model to be built and more accurate decisions to be made. Further, CoCoSo can handle complex problems more easily and efficiently [13].
- The combination of IF-AHP, IF-DEMATEL, and CoCoSo can be easily applied not only for listing ED alternatives but also for handling real-world problems in different disciplines. Thanks to this combination, the alternatives are ranked and a projection can be obtained for decision-makers about potential improvements in which criteria will affect the ranking. In this way, a roadmap can be created.

The organization of the research is designed under five sections. The following section presents an in-depth overview of the related work on the topic. It handles the current knowledge from two aspects; while one is on the previous efforts of performance assessment of health facilities during outbreaks, the other is regarding outbreak readiness assessment by MCDM methods. The third section provides the mathematical background of the proposed methodology. The fourth section introduces the numerical results obtained from the case study application. The final section provides a general conclusion, limitations, and future research agenda.

## 2. Related Work

This section, in which the evaluation of ED performance during the COVID-19 outbreak will be discussed in detail, is organized under three main subsections. In the first of these, the performance or preparation of health facilities in case of epidemic disasters is discussed by looking at the issue with a slightly more inclusive perspective and with the aid of models created from the medical perspective. The second part includes the analysis of MCDM methods used in performance evaluation for epidemic situations, which form the basic framework of this article. In the last section, the main contributions and innovations of the article are provided.

### 2.1. Performance Assessment of Health Facilities during Outbreaks

Due to the COVID-19 pandemic, healthcare facilities have taken significant steps to develop preventive measures, testing procedures, and emergency response plans [14]. Those in a decision-making position in health are implementing new health and safety plans to control the spread and adapt people to the new normal. Hospitals are at the forefront of the health institutions that bear the greatest burden of the epidemic. In these facilities, the EDs, which are the most prominent and undoubtedly provide first aid to the patient, are the units that create prevention measures and preparations in the pandemic. In this regard, Garcia-Lopez et al. [15] developed a hospital readiness checklist to assess the hospital’s ability to respond to the COVID-19 pandemic. They interviewed 32 experts with high-level experience in hospital management and epidemic prevention to create several preparatory projects. However, in many studies in the literature, epidemic prevention measures are discussed from different perspectives [16,17,18,19,20,21,22,23,24,25]. Focusing on medicine and healthcare personnel, Griffin et al. [26] planned a hospital readiness guideline based on a critical care perspective, focusing on multiple areas, intensive care unit (ICU) surge capacity, concurrent infection control, clinical operational challenges, ethics, staffing, and staff health protection. In addition, Gul and Yucesan [16] proposed a preparedness ranking model for Turkish hospitals in response to COVID-19. A total of 99 criteria for the hospital’s epidemic prevention efforts are considered for this multi-criteria model. In addition to the papers mentioned above, many researchers have contributed to the outbreak prevention studies for healthcare facilities [24,27,28,29,30,31]. Boufkhed et al. [27] focused on the preparedness and capacity of palliative care services in Middle Eastern and North African countries. Mukhtar [28] developed a proactive infection prevention measure approach for outbreak control in Pakistan. That study presented comprehensive anti-epidemic measures in Pakistan and highlighted the importance of personal protection, waste disposal, and route planning. Gupta and Federman [29] studied hospital readiness based on the experience of the Veterans Affairs Connecticut healthcare system. AlTakarli [30] suggested a pandemic preparedness model for China. They highlighted that efforts to develop a resilient system against infectious diseases are one of the country’s most important priorities. Noh et al. [31] recommended strategies for safe hospital operations in Korea. They presented a Swiss cheese model to evaluate hospitals’ ability against epidemics.

### 2.2. Outbreak Readiness Assessment by MCDM Methods

As a result of the increasing disaster events around the world in recent years, the science of decision-making is of great importance in combating these events. Because real-world decision-making problems are complex and challenging, especially when faced with unstructured problems, there is no way to solve them by building mathematical models. MCDM is preferred more strongly than mathematical models because ED performance assessment problems include qualitative and quantitative criteria together, the expert’s perspective is essential to handle the criteria, it is not possible to determine the relationship between the criteria mathematically, and it provides the opportunity to determine local and global significance scores [12]. MCDM is an operations research sub-field that can be used very frequently and effectively in solving the above-mentioned problems. With these methods, decision-makers can manage a robust decision-making process to make decisions. MCDM generally includes three main stages: establishing evaluation criteria, calculating criterion weights, and prioritizing alternatives [32]. However, in the global COVID-19 pandemic, problems such as many epidemic prevention decisions, preparedness assessment of health facilities, and selection of the most appropriate struggling type are typical MCDM problems [16,31,33,34,35,36,37,38,39]. In recent years, many studies using MCDM-based approaches have been published to evaluate the preparedness of health facilities in the event of a COVID-19 outbreak or any disaster [16,40]. For example, Ortiz-Barrios et al. [33] proposed a hybrid model of analytic hierarchy process (AHP) and technique for order performance by similarity to ideal solution (TOPSIS) to assess sales departments’ level of COVID-19 preparedness. In a similar AHP-TOPSIS model, Hezam et al. [41] prioritized vaccine alternatives using neutrophilic set theory. Gül and Yücesan [16] proposed the interval-valued global fuzzy set-based TOPSIS model to measure the readiness performance of Turkish hospitals providing tertiary care. Moheimani et al. [34] used a rough set theory to classify the level of preparedness of hospitals. Again, in recent studies, Saner et al. [36] studied the evaluation of the disaster preparedness of hospitals with the Bayesian best–worst method (Bayesian BWM)-VIekriterijumsko KOmpromisno Rangiranje (VIKOR) integrated approach. Ortiz-Barrios et al. [40] and Ortiz-Barrios et al. [37] presented MCDM-based models for hospitals’ disaster preparedness by presenting case studies from Colombia and Turkey.

While most existing models deal with the problem in hospitals, this study focused directly on emergency services. In addition, from a methodological point of view, there are differences between the existing models and the models briefly explained above. The existing model has been brought together for two ultimate purposes. The first is to determine the importance level of the criteria, taking into account the interdependence, and the second is to determine the performance of emergency services in Turkey. AHP, the most frequently applied MCDM method in determining criterion weights in a traditional decision-making problem, is handled under intuitionistic fuzzy sets. The intuitionistic fuzzy set concept was first proposed by Atanassov [42]. It is a generalization of Zadeh’s [43] classical fuzzy set theory. The fuzzy set reflects uncertainty and uncertainty well in computational analysis. While Zadeh’s fuzzy set proposes only a membership function, Atanassov’s intuitionistic fuzzy set also provides a non-membership function. Therefore, we combined it with AHP to assign weights based on linear dependence on emergency service performance assessment criteria under COVID-19. Linear dependency is related to the hierarchical structure in AHP. The hierarchy shows the relationship between the elements of a level and the level elements immediately below it. This relationship continues to the lowest levels of the hierarchy and determines how each element is at least indirectly related to each other [44]. When comparing items at each level, a decision-maker should only compare according to the contribution of lower-level items to higher-level items. Alongside IF-AHP, we merged IF-DEMATEL to our proposed approach to identify interrelationships between these criteria and feedback. DEMATEL was originally developed as an MCDM and graph theory-based network analysis tool [45,46,47]. It is based on the causal relationship within a network and distinguishes the criteria under “cause” and “effect” groups. It is combined with the intuitionistic fuzzy set to reflect the uncertainty encountered in making judgments in DEMATEL. IF-DEMATEL has been applied to important health problems such as that by Ocampo and Yamagishi [48]. When the available information is scanned, it can easily be observed that combining AHP-DEMATEL under the intuitionistic fuzzy concept is indeed new. Considering the importance of emergency service preparedness during COVID-19, applying this new approach in emergency and disaster management is useful and merits the current knowledge. CoCoSo was employed for the final stage of the model. Although CoCoSo has been applied to several MCDM problems [12,13,49], its application to the issue of how hospital emergency services will perform in preparation for a COVID-19-like disaster is still limited. Therefore, there is a gap in the literature in this regard. To remedy this gap, such a model is proposed.

## 3. Proposed Methodology

A six-step approach is proposed to evaluate the emergency department response during the COVID-19 outbreak, identify the intervention points, and design improvement plans to increase their response in the presence of COVID-19 (Figure 1). An explanation of the suggested methodology is provided below:

***Step 1. Creation of an expert team*:** A set of decision-makers is selected considering their background in ED management. The experts are expected to provide information on which factors may affect the performance of these units throughout the current pandemic. Furthermore, they will be asked to carry out paired judgments to define both the importance and influence of the decision elements.
***Step 2. Structuring the ED performance evaluation model:*** The decision model is then defined by including ED performance criteria and sub-criteria from the pertinent reported literature, legal healthcare framework, and decision-makers’ recommendations.
***Step 3. Computation of criteria and sub-criteria relative priorities considering uncertainty:*** IF-AHP method is later applied to derive the relative weights of criteria and sub-criteria while modelling the uncertainty and vagueness of human thought when performing the paired judgments. The IF-AHP results will lay the groundwork for the proposal of short-term improvement interventions that is highly needed in a rapidly evolving pandemic.
***Step 4. Appraisal of cause–effect interrelations among criteria and sub-criteria considering uncertainty:*** IF-DEMATEL is applied to detect significant interdependence and feedback among criteria/sub-criteria whilst representing the expected uncertainty of decision-makers when eliciting the influence comparisons in the model. IF-DEMATEL goes beyond measuring the strength of these interrelations, so that the main drivers of long-term plans can be easily defined.
***Step 5. Calculation of the ED performance index and ranking derivation*:** In this phase, CoCoSo is employed for computing a performance index for each ED. Following this, the emergency care units are ranked from the highest to the lowest value of this index to discriminate those with high performance $\left(M_{i}\;\geq 0.75\right)$ as well as the EDs with an urgent need for improvement $\left(M_{i}\leq 0.25\right)$.
***Step 6. Identification of intervention points and creation of intervention plans per each ED:*** Detect the sub-criteria most contributing to a poor response by EDs against the COVID-19 outbreak and delineate focused improvement plans targeting upgraded performance.

### 3.1. Intuitionistic Fuzzy Analytic Hierarchy Process (IF-AHP)

The theory of fuzzy sets was first proposed by Zadeh [43] and allows us to introduce the uncertainty of a decision-maker based on precise concepts. Later, Atanassov [42] improved this work by considering the hesitancy degree, which denotes the proficiency of each expert in a particular decision-making context. This new approach is called the intuitionistic fuzzy set (IFS) theory and comprises the following foundations:

***Concept of an intuitionistic fuzzy set:*** IFS is an extension of fuzzy sets (IF) where *X* represents a set in the universe of discourse. An intuitionistic set “*A*” is given by Equation (1) [50,51].

(1) $$A=\{\;\langle x,\;A\;\left(\mu_{A}\left(x\right),\;v_{A}\left(x\right)\right)\rangle |x\in X\;\}$$

Here, $\mu_{A}\left(x\right):X\;a\;\left[0,1\right]$ denotes the degree of membership whilst $v_{A}\left(x\right):X\;a\;\left[0,1\right]$ represents the degree of non-membership. The applicable mathematical properties for each x∈X are in Equations (2) and (3). Specifically, the hesitancy degree is given by Equation (3).

(2) $$0\leq \mu_{A}\left(x\right)+v_{A}\left(x\right)\leq 1$$

(3) $$\pi_{A}\left(x\right)=1-\mu_{A}\left(x\right)-v_{A}\left(x\right),\;x\in X$$

***Concept of defuzzification process in IFS:*** Defuzzification involves converting fuzzy quantities into crisp values for further estimation of the relative priorities and interdependencies of criteria and sub-criteria for the application described here. In this regard, Attanassov et al. [42] postulated a defuzzification for the IFS as stated in Equations (4) and (5). First, the defuzzification operator $C_{\varphi }$ (Equation (4)) allows converting an IFS into a classical fuzzy set (FS) followed by the evaluation of the FS via a defuzzification approach. Although different score functions can be utilized for transforming the IFS into crisp values [52], using the operator $C_{\varphi }$ has been proven to be more useful in practical scenarios due to its mathematical simplicity. Today’s healthcare administrators aim to employ robust and rigorous decision-making approaches involving single equations or few steps to follow as exemplified with $C_{\varphi }$. For example, Aicevarya Devi et al. [53] proposed a converting intuitionistic fuzzy into a crisp score (CIFCS) algorithm to deal with the defuzzification activity, but its application is not recommended in this context given the significant mathematical effort involved in MCDM models with many decision elements. On a different tack, Büyüközkan et al. [54] employed the intuitionistic fuzzy entropy method but did not provide further mathematical validation supporting its implementation, which in turn was specified by Atanassov et al. [42] regarding $C_{\varphi }$.

(4) $$C_{\varphi }\left(A\right)=\left\{x,\mu_{A}\left(x\right)+\varphi \pi_{A}\left(x\right),v_{A}\left(x\right)+\left(1-\varphi \right)\pi_{A}\left(x\right),x\epsilon X\right\}\;\mathrm{with}\;\varphi \epsilon \left[0,1\right]$$

Here, $C_{\varphi }\left(I\right)$ is a classical fuzzy subset whose membership degree is defined by the equation:

(5) $$\mu_{\varphi }\left(x\right)=\mu_{A}\left(x\right)+\varphi \pi_{A}\left(x\right)$$

The solution of the minimization problem for a Euclidean separation *d*, i.e.,$\underset{\varphi \epsilon \left[0,1\right]}{\min}d(C_{\varphi }\left(A\right),\;A)$, is achieved when φ=0.5. The fuzzy set $C_{0.5}\left(A\right)$ is now denoted by the membership expression $\mu \left(x\right)=\frac{1}{2}\left(1+\mu_{A}\left(x\right)-v_{A}\left(x\right)\right)$. Finally, the center of gravity is suggested for supporting the defuzzification process [55].

After explaining the IFS logic underpinning the inclusion of uncertainty and hesitancy in the decision-making model, we can now introduce the IF-AHP method, which has been selected for weighting the criteria and sub-criteria defining the ED readiness during the COVID-19 pandemic [56]. The step-by-step IF-AHP procedure is presented as follows:

***Phase 1—*Identification of criteria and sub-criteria defining the ED readiness against the COVID-19 outbreak:** In this phase, a decision-making group is formed to provide information on the aspects directly contributing to ED readiness during the current pandemic times. Moreover, applicable health regulations and pertinent scientific literature are consulted to extract other decision elements to be considered in the model.

***Phase 2—*Pairwise comparisons:** The decision-making team is asked to perform paired judgments between criteria and sub-criteria considering the following intuitionistic fuzzy evaluation scale: much less relevant (0.27, 0.33, 0.40), less relevant (0.27, 0.13, 0.60), equally relevant (0.02, 0.18, 0.80), more relevant (0.13, 0.27, 0.60), and much more relevant (0.33, 0.27, 0.40).

***Phase 3—*Estimation of the importance weights per expert:** The scale of Boran et al. [51] is used for determining the knowledge degree of experts. The higher the level of experience of the decision-maker, the higher the relative priority. The corresponding triangular intuitionistic fuzzy numbers considered in [43] are as follows: very irrelevant (0.10, 0.80, 0.10), irrelevant (0.25, 0.60, 0.15), moderately relevant (0.50, 0.40, 0.10), relevant (0.75, 0.20, 0.05), and very relevant (0.90, 0.05, 0.05). In this regard, $D_{k}=\left(\mu_{k},v_{k},\pi_{k}\right)$ has been denoted as an intuitionistic number for the fuzzy importance evaluation of a *k*th expert. The $\omega_{k}$ indicates the relative priority of the *k*th expert (Equation (6)).

(6) $$\omega_{k}=\frac{\left(\mu_{k}+\pi_{k}\left(\mu_{k}/\left(\mu_{k}+v_{k}\right)\right)\right)}{\sum_{k=1}^{t}\left(\mu_{k}+\pi_{k}\left(\mu_{k}/\left(\mu_{k}+v_{k}\right)\right)\right)}$$

***Phase 4—*Derive the aggregated intuitionistic fuzzy decision-making matrix**$R^{\left(k\right)}$**:** Here, $R^{\left(k\right)}={\left(r_{ij}^{\left(k\right)}\right)}_{mxn}$ denotes an intuitionistic fuzzy decision matrix estimated based on the judgments of *K* decision-makers. Xu [57] and Ar et al. [58] employed a generalized intuitionistic fuzzy weighted average (IFWA) operator to facilitate the aggregation of the comparisons (Equations (7) and (8)).

(7) $$r_{ij}=IFWA_{\omega }=\left(r_{ij}^{\left(1\right)},r_{ij}^{\left(2\right)},\ldots .,r_{ij}^{\left(t\right)}\right)=\omega_{1}r_{ij}^{\left(1\right)}⨁\omega_{2}r_{ij}^{\left(2\right)}⨁\ldots ⨁\omega_{t}r_{ij}^{\left(t\right)}$$

(8) $$IFWA_{\omega }=\left(1-\prod_{k=1}^{t}{\left(1-\mu_{ij}^{\left(k\right)}\right)}^{\omega_{k}},\prod_{k=1}^{t}{\left(v_{ij}^{\left(k\right)}\right)}^{\omega_{k}},\prod_{k=1}^{t}{\left(1-\mu_{ij}^{\left(k\right)}\right)}^{\omega_{k}}-\prod_{k=1}^{t}{\left(v_{ij}^{\left(k\right)}\right)}^{\omega_{k}}\right)$$

where $r_{ij}=\left(\mu_{ij},v_{ij},\pi_{ij}\right)$.

***Phase 5—*Consistency assessment:** The experts need to be consistent when making the comparisons, so that the resulting relative priorities of criteria and sub-criteria can be a reliable framework for hospital readiness evaluation. The consistency ratio (CR) (Equation (9)) proposed by Saaty [44] has been adopted for detecting discrepancies in the aggregated intuitionistic fuzzy decision matrixes.

(9) $$CR=\frac{\left(\left(\lambda_{max}-n\right)/\left(n-1\right)\right)}{RI}$$

In Equation (9), *n* denotes the number of criteria and sub-criteria integrating the matrix. If the resulting CR is lower than 0.10, the matrix is considered consistent; otherwise, the experts should revise and update their judgments before further estimation of relative global and local priorities [59].

***Phase 6—*Computation of the intuitionistic fuzzy weights:** Once consistency has been achieved in all the matrixes, we proceed with the calculation of intuitionistic fuzzy priorities for criteria and sub-criteria by applying Equations (10) and (11). The priorities should be standardized to ensure $\sum w_{i}=1$.

(10) $${\overset{=}{w}}_{i}=-\frac{1}{nln2}\left(\mu_{i}ln\mu_{i}+v_{i}lnv_{i}-\left(1-\pi_{i}\right)\ln\left(1-\pi_{i}\right)-\pi_{i}ln2\right)$$

(11) $$w_{i}=\frac{1-{\overset{=}{w}}_{i}}{n-\sum_{i=1}^{n}{\overset{=}{w}}_{i}}$$

### 3.2. Intuitionistic Fuzzy Decision-Making Trial and Evaluation Laboratory (IF-DEMATEL)

The DEMATEL method has been used in several decision-making problems for appraising the interdependence and feedback among the criteria/sub-criteria of a network [45,46,47]. Nonetheless, in some occasions, the crisp scale is inadequate to estimate the vague interrelations, thereby making the model less realistic and applicable to the practical context. A merge with IFS logic is then suggested to deal with this problem [60,61]. IFS concepts have been widely introduced in the previous section and underpin the IF-DEMATEL algorithm as illustrated below.

***Phase 1—*Paired comparisons:** In IF-AHP, the decision-makers assess the linear dependency of criteria and sub-criteria with respect to their adjacent decision elements in the same hierarchy level. Nonetheless, the hospital’s daily routine and the related reported literature have also revealed the presence of significant interactions among healthcare services and indicators. Therefore, analyzing these interrelations can provide significant outputs on hospital disaster preparedness while laying the groundwork for the design of medium- and long-term intervention plans. In this respect, the IF-DEMATEL entails the following IFS evaluation scale: no influence [0.1, 0.9], low influence [0.35, 0.6], medium influence [0.5, 0.45], high influence [0.75, 0.2], and very high influence [0.9, 0.1]. The decision-makers are also required to perform the judgments pairwise in the search of elucidating the cause and effect groups. The degree of hesitation is calculated using Equation (3) and an initial intuitionistic fuzzy matrix $\tilde{Z_{k}}={\left[\tilde{z_{ij}^{k}}\right]}_{nxn}$ is formed with the comparisons.

***Phase 2—*Defuzzification:** In this phase, the intuitionistic fuzzy matrixes are defuzzified by first transforming $\tilde{Z_{k}}$ into a classical fuzzy subset following the formula $\mu \left(x\right)=\frac{1}{2}\left(1+\mu_{I}\left(x\right)-v_{I}\left(x\right)\right)$. Then, the resulting subsets are allocated to a triangular fuzzy number (TFN) by implementing the Equations (12) and (13) [48]. Here, $\mu \left(\tilde{x}\right)$ denotes the membership function value while $\tilde{x}$ symbolizes the respective defuzzified value. Further, *l*, *m*, and *u* are the parameters of TFNs. A matrix $Z_{k}={\left[z_{ij}^{k}\right]}_{nxn}$ with the subsequent crisp numbers is derived accordingly, considering *k* (*k* = 1, 2, …, *K*) as the *k*th expert in the decision-making group.

(12) $$\mu \left(\tilde{x}\right)=\left(\tilde{x}-l\right)/\left(m-l\right)$$

(13) $$\tilde{x}=l+\mu \left(\tilde{x}\right)\left(m-l\right)$$

***Phase 3—*Aggregated initial direct-relation matrices:** In this step, the aggregated crisp matrix $Z={\left[z_{ij}\right]}_{nxn}$ is achieved by implementing Equation (14).

(14) $$z_{ij}=\frac{1}{l}\sum_{k=1}^{l}z_{ij}^{k},\;\;\;\;\;\;i,j=1,2,\ldots ,n$$

***Phase 4—*Convergence index computation:** It is also important to validate the reliability of the results obtained from IF-DEMATEL to avoid possible errors in expert decision-making. Nevertheless, most DEMATEL applications ignore biases and accept that the information is trustworthy. Hence, we propose the use of the convergence index for assessing the internal consistency among paired judgments. This ratio allows us to define if the experts were accurate in making their comparisons. The integration matrix of *n* and *n* − 1 decision-makers is denoted by $g_{c}^{ijp}$ and $g_{c}^{ij\left(p-1\right)}$(Equation (15)). The participants are deemed consistent if this ratio is found to be less or equal to 0.05.

(15) $$\frac{1}{n\left(n-1\right)}\sum_{i=1}^{n}\sum_{j=1}^{n}\frac{\left|g_{c}^{ijp}-g_{c}^{ij\left(p-1\right)}\right|}{g_{c}^{ijp}}$$

***Phase 5—*Normalization of the initial direct relation matrix (*N*):** After verifying the consistency of the comparisons, the matrix $N={\left[N_{ij}\right]}_{nxn}\;$is obtained by normalizing the average matrix (Equations (16) and (17)). Here, *s* is the norm used for achieving *N*.

(16) $$N=s^{-1}Z$$

(17) $$s=max\left(\sum_{j=1}^{n}z_{ij}\;,\sum_{i=1}^{n}z_{ij}\;\right)$$

***Phase 6—*Estimation of the total influence matrix (*T*):** The *T* array, considering the normalized (*N*) and identity (*I*) matrixes (Equation (18)), reveals the direct and indirect effects of each criterion/sub-criterion, thereby supporting the identification of dispatchers and receivers. From this matrix, it is possible to calculate the “Prominence” (D + R) of each decision element, which denotes the strength of interdependencies emanating from and received by a specific criterion/sub-criterion. Thereby, decision-makers can elucidate how critical its role is regarding the network goal. In parallel, “Relation” (D − R), another parameter of importance in medium- and long-term decisions, denotes the nature of criteria/sub-criteria in ED disaster preparedness. Specifically, if $d_{i}-r_{j}>0$, the decision element is then categorized as “dispatcher”; therefore, it is of influencing essence and can be prioritized for upgrading ED performance in the presence of the COVID-19 pandemic waves. Otherwise, if $d_{i}-r_{j}<0$, the sub-criterion/criterion is a “receiver” and it is hence influenced by others in the network. It is good to highlight that *d*_i_ is the *i*th row sum of effects that a specific element exerts on the others (Equation (19)), whereas *r_j_* is the *j*th column sum of influences received by a criterion/sub-criterion in the ED disaster preparedness model (Equation (20)).

(18) $$T=N{\left(I-N\right)}^{-1}$$

(19) $$D={\left[d_{i}\right]}_{nx1}={\left(\sum_{j=1}^{n}t_{ij}\right)}_{nx1}={\left(t_{i}\right)}_{nx1}$$

(20) $$R={\left[r_{j}\right]}_{1xn}={\left(\sum_{i=1}^{n}t_{ij}\right)}_{1xn}={\left(t_{j}\right)}_{1xn}$$

***Phase 7—*Impact-Digraph Map (IDM):** The dataset (D + R, D − R) is finally plotted to derive the significant interrelations among criteria/sub-criteria. At this point, it is necessary to calculate a threshold θ per matrix by using Equation (21). All the $t_{ij}$ over this reference value are considered significant and are then incorporated into the IDM.

### 3.3. Combined Compromise Solution (CoCoSo)

**Figure 2:**
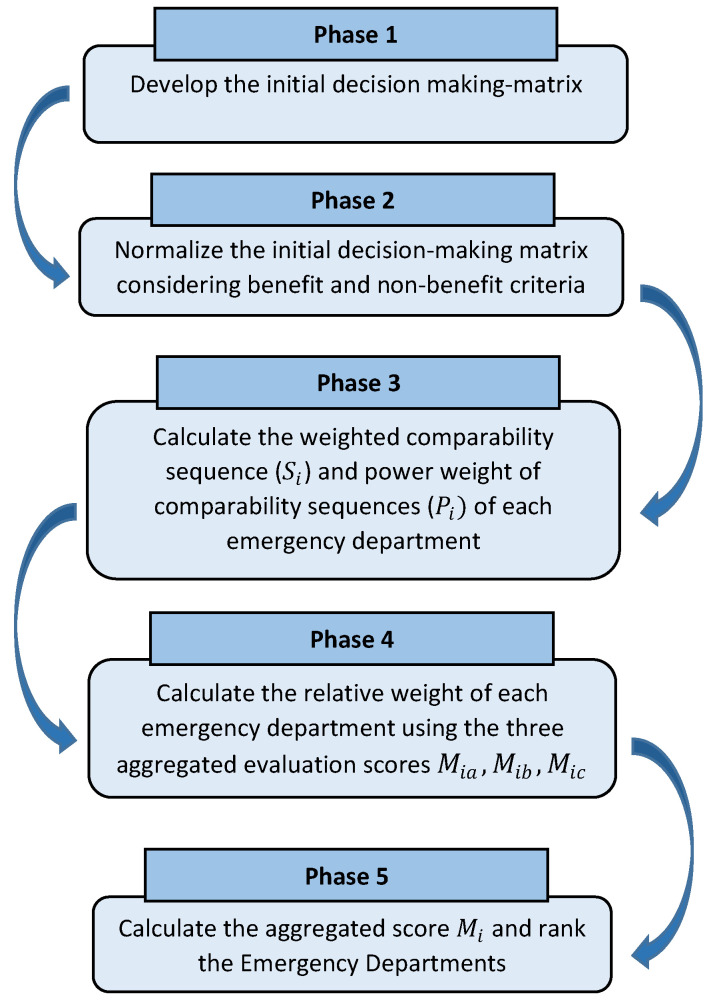
The step-by-step procedure of the CoCoSo method.

The CoCoSo (combined compromise solution) method is a novel multi-criteria method presented by Yazdani et al. [13]. It comprises three well-known MCDM methods and is based on compromise solutions such as TOPSIS and measurement alternatives and ranking according to compromise solution (MARCOS). Many MCDM methods have been proposed in the literature and are still being developed further. Here, the feature that brings CoCoSo to the forefront and triggers our application for the problem is that the method combines three different strategies and gives an aggregated ranking with the features of all three: simple additive weighting (SAW), multiplicative exponentially weighting (MEW), and weighted aggregate sum product assessment (WASPAS). In the literature, it has been stated that most combined methods or new approaches that include the features of several methods give good results [13]. It differentiates from MARCOS, which is based on utility functions on which decision preferences are defined. It has similar features to TOPSIS. While MARCOS is based on determining the relationship between alternatives and reference values (ideal and anti-ideal solutions), CoCoSo obtained a compromise solution by combining the utility values of each alternative using an aggregation function [62]. Rank reversal or ranking irregularities (that is, the ordering of alternatives may change if a new option is added to the presented set of options or an old option is deleted or changed from it) is undoubtedly one of the most important handicaps of many MCDM methods [63,64,65]. It has been evaluated in the literature to be a normal phenomenon [65]. Therefore, in this study, an MCDM technique such as CoCoSo was also preferred purely because of its benefits (outlined above) and ease of integration with other fuzzy-based MCDM methods (like IF-AHP and IF-DEMATEL). This method helps to calculate an aggregated score representing the ED disaster preparedness against COVID-19 while supporting decision-makers in elucidating the weakness and strengths of each emergency unit [12,49]. The CoCoSo procedure is illustrated in Figure 2.

***Phase 1—*Establish the initial decision matrix:** An indicator is first defined for each sub-criterion and then measured in each participant ED $\left(m_{ij}\right)$. The collected data is finally arranged in matrix *M* (Equation (21)), where relative global priorities derived from the IF-AHP method are also incorporated. In Equation (21), *I* denotes the alternative ED, whilst *j* represents the sub-criterion.

(21) $$M=\left[m_{ij}\right]$$

***Phase 2—*Normalization:** Normalize *M* by applying Equations (22) and (23). The resulting matrix *R* = $\left[r_{ij}\right]$ compiles all the normalized values $\left(r_{ij}\right)$, considering if the sub-criterion is of benefit (Equation (22)) or non-benefit (Equation (23)) nature.

(22) $$r_{ij}=\frac{m_{ij}-\underset{i}{\min}m_{ij}}{\underset{i}{\max}m_{ij}-\underset{i}{\min}m_{ij}}\;\mathrm{for}\;\mathrm{benefit}\;\mathrm{subcriteria}$$

(23) $$r_{ij}=\frac{\underset{i}{\max}m_{ij}-m_{ij}}{\underset{i}{\max}m_{ij}-\underset{i}{\min}m_{ij}}\;\mathrm{for}\;\mathrm{non}-\mathrm{benefit}\;\mathrm{subcriteria}$$

***Phase 3—*Estimation of weighted comparability** $\left(S_{i}\right)$ **and power-weighted comparability**$\left(P_{i}\right)$**:** $S_{i}$ and $P_{i}$ are computed for each participant ED by applying the Equations (24) and (25). Here $w_{j}$ symbolizes the relative weight of the sub-criterion *j*.

(24) $$S_{i}=\sum_{j=1}^{n}w_{j}r_{ij}$$

(25) $$P_{i}=\sum_{j=1}^{n}r_{ij}{}^{w_{j}}$$

***Phase 4—*Computation of aggregated appraisal scores:** $M_{ia},\;M_{ib},M_{ic}$ are estimated for each participant emergency care unit *i* via implementing Equations (26)–(28), respectively. These formulas are based on the $S_{i}$ and $P_{i}$ scores calculated for each alternative.

(26) $$M_{ia}=\frac{P_{i}+S_{i}}{\sum_{i=1}^{m}\left(P_{i}+S_{i}\right)}$$

(27) $$M_{ib}=\frac{S_{i}}{\underset{i}{\min}S_{i}}+\frac{P_{i}}{\underset{i}{\min}P_{i}}$$

(28) $$M_{ic}=\frac{\lambda \left(S_{i}\right)+\left(1-\lambda \right)\left(P_{i}\right)}{\lambda \underset{i}{\max}S_{i}+\left(1-\lambda \right)\underset{i}{\max}P_{i}}$$

***Phase 5—*Ranking and design of improvement interventions:** Order the participant EDs from the highest to the lowest $M_{i}$ value (Equation (29)). $M_{i}$ represents the emergency department performance index (EDPI), which will allow decision-makers to establish the overall response that these units may evidence in the presence of new COVID-19 waves and similar seasonal respiratory diseases. Then, define strategies on each weak sub-criterion, so that ED preparedness can be increased when addressing COVID-19 waves.

(29) $$M_{i}=\sqrt[{3}]{\left(M_{ia}M_{ib}M_{ic}\right)}+\frac{1}{3}\left(M_{ia}+M_{ib}+M_{ic}\right)$$

## 4. Results

The Turkish healthcare system has been fighting against the COVID-19 pandemic. As a result, the EDs have evidenced accessibility problems including overcrowding, lengthy waiting times, and a high percentage of no-shows [16]. In fact, in January 2022, the Ministry of Health counted 74,266 cases in one day, the highest infection rate in the country since the beginning of the pandemic [66]. One of the cities with the highest rate of hospitalization and bed occupancy in hospitals is Istanbul, which has motivated the adoption of more stringent measures. The described scenario is then an incentive to:

- measure the performance of EDs and create strategies to increase their response to the ongoing sanitary crisis;
- identify influential factors that can be prioritized by health authorities when designing the ED policies at a national level;
- pinpoint the limitations of each ED and implement focused improvement plans.

The following subsections describe the implementation of the suggested MCDM hybrid approach to address the aforementioned considerations.

### 4.1. The Expert Team

Adequately selecting a team of decision-makers is important to determine the relevance and influence of the performance criteria/sub-criteria considered in the response of EDs against the COVID-19 emergency. The experts were selected considering three inclusion criteria: (i) professional background in emergency department management, (ii) more than 10 years of experience in the healthcare sector, and (iii) have participated in projects pursuing upgraded response of EDs during disastrous situations. The resulting sample size (*n* = 6 experts) was derived considering *N* = 6 experts satisfying the above-mentioned criteria, with *p* = 0.5, q = 0.5, confidence level = 95%, and E = 5%. Taking into account the above, the following experts were chosen:

- Three ED supervisors (DM1, DM2, and DM3): They were linked to public sector hospitals and presented in-depth knowledge in the field of hospital emergency services. Moreover, they had a significant background represented by a trajectory of more than 15 years in the healthcare industry.
- Two healthcare quality auditors (DM4 and DM5): The two experts were chosen for their roles as spokespersons involved in radical changes improving emergency care. Further, they were related to the implementation of healthcare government policies and can be thereby useful for devising improvement plans and contributing to the optimal development of emergency services in public hospitals.
- One senior academician (DM6): She currently has a high level of experience in the application of multi-criteria decision-making techniques. Moreover, she had a constant enrollment in projects related to the health sector, thereby knowing the main weaknesses of the emergency care services in public hospitals.

### 4.2. The ED Performance Evaluation Model

**Figure 3:**
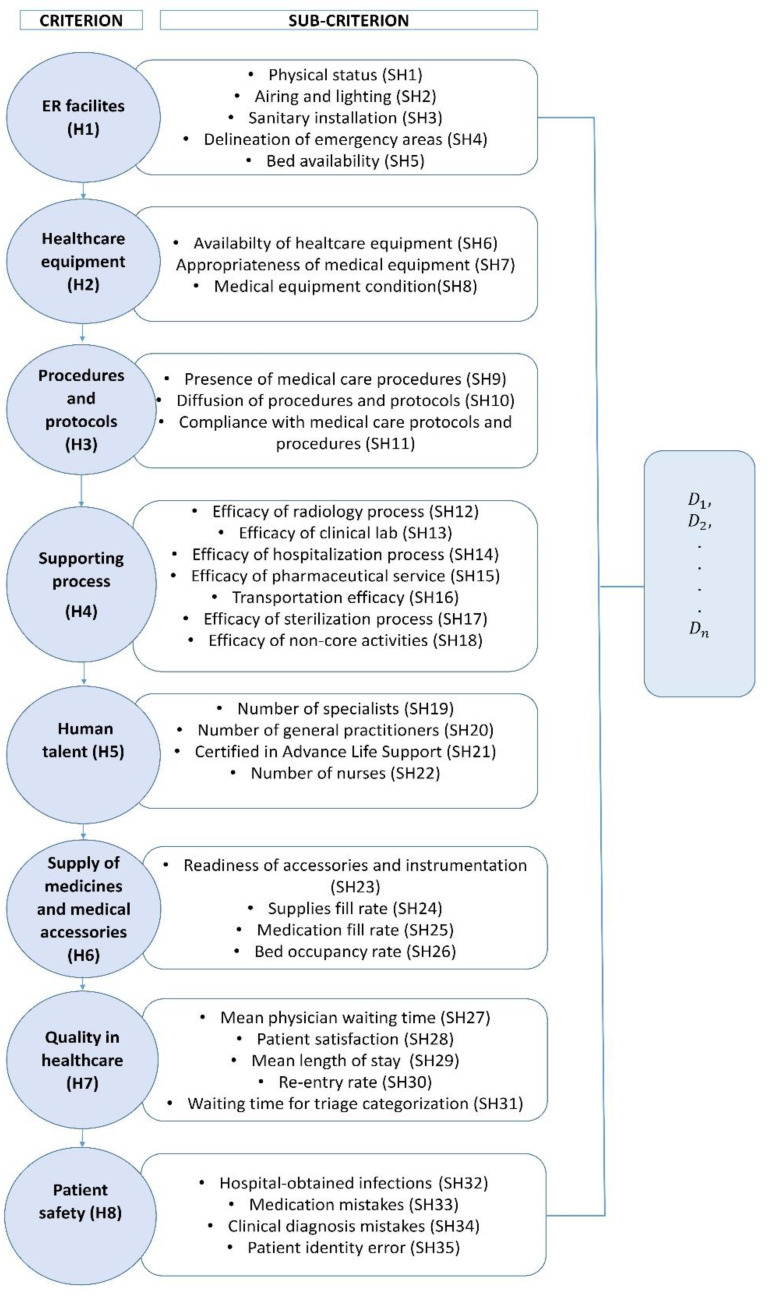
Decision network for performance evaluation of emergency care centers during the COVID-19 waves.

A total of 8 criteria and 35 sub-criteria were finally identified as performance drivers of emergency centers in Turkish public hospitals. This is the result of the interactions with experts’ feedback, the evidence from the scientific literature, and the pertinent healthcare regulations in the COVID-19 era. Specifically, phone calls were performed by the researchers to collect the criteria and sub-criteria that the experts recommended including in the performance evaluation model. Further, Scopus and Web of Science databases were consulted to extract the decision elements that similar studies had considered in previous applications. Likewise, “clause 5: provision of emergency health services in the national territory”, clause 9 “optimal functioning of emergency centers in hospitals”, and clause 15 “written duties and responsibilities of all trained personnel in the emergency center” were analyzed to identify the performance factors separately measured and supervised by the health authorities concerning the emergency care service. The legal framework was deemed in this study to validate the initial list of criteria and sub-criteria derived from the expert team and the reported literature. Thereby, it was possible to ensure that the resulting model is pertinent to the real healthcare scenario and the COVID-19 context. These information sources then provided a clear basis for the aspects to be deemed when implementing improvement plans that upgrade the response of emergency care services against new waves of COVID-19 and other similar respiratory epidemics/pandemics. The identified decision elements (sub-criteria) were then grouped into eight clusters (criteria) to obtain a final version of the ED performance evaluation model (Figure 3).

Complementary to the above, each decision element is further explained. The first criterion is “ER Facilities (H1)”, which is composed of five sub-criteria: Physical status (SH1), Airing and lighting (SH2), Sanitary installations (SH3), Delineation of emergency areas (SH4), and Bed availability (SH5). More specifically, the *“Physical status”* denotes the current status of the emergency department wards in terms of well-being, operability, and patient safety. Another important factor is the *“Airing and lighting”*, which evidences the compliance of air conditioning and lighting standards that are essential to provide emergency care during a pandemic. Undoubtedly, *“Sanitary installations”* is also a criterion to be considered by policymakers since it measures the availability of cleaning areas within the emergency wards. On the other hand, *”Delineation of emergency areas”* examines whether the waiting areas, triage rooms, and observation lounges are delimited and demarcated to avoid patient flow problems. Likewise, *“Bed availability”* has gained increasing popularity during the COVID-19 outbreak given the multiple shortages reported by healthcare systems [67,68], and it should be therefore included in this analysis.

The second criterion in the network model is “Healthcare equipment (H2)”, which is relevant for diagnosis/treatment support while collecting patient data from admission time to discharge. In this regard, some aspects must be taken into account. Firstly, “*Availability of healthcare equipment*” denotes the proportion of medical devices that is ready for use within the daily emergency care routine. On a different note, “*Appropriateness of medical equipment*” evaluates if these devices accomplish the minimum operability standards established by the healthcare authorities and are pertinent to the emergency care context during a pandemic. Another important aspect in this decision cluster is the “*Medical equipment condition*”, which verifies the correct operability of these machines according to the design specifications including the characteristics of COVID-19 patients.

The third criterion “Procedures and protocols (H3)” denotes the healthcare guidelines to be followed by physicians, nurses, and administrative staff for supporting ED decision-making during the COVID-19 outbreak. Three elements should be taken into account: “*Presence of medical care procedures*”, “*Compliance with medical care protocols and procedures*”, and “*Diffusion of procedures and protocols*”. First, “*Presence of medical care procedures*” evaluates whether the hospital’s standard operating procedures have been included in the ED quality management system. The second sub-criterion “*Diffusion of procedures and protocols*” determines whether these standard operating procedures have been disseminated and explained to the ED workers before implementation. Finally, “*Compliance with medical care protocols and procedures*” establishes whether all the protocols and standards established in the QMS are being correctly applied in the ED’s daily operation.

Another factor considered within this decision framework is “Supporting processes (H4)”, which has been subdivided into seven aspects: “*Efficacy of radiology process*”, “*Efficacy of clinical lab*”, “*Efficacy of hospitalization process*”, “*Efficacy of pharmaceutical service*”, “*Transportation efficacy*”, “*Efficacy of sterilization process*”, and “*Efficacy of non-core activities*”. The first sub-criterion evaluates the time invested by the radiology department to provide diagnostic images of the patient admitted to the emergency center. The second sub-criterion analyzes the time used by the laboratory to derive the test results supporting the medical diagnoses and treatments associated with ED patients. Likewise, “*Efficacy of hospitalization process*” measures the average waiting time for a hospitalization bed, while “*Efficacy of pharmaceutical service*” denotes the time spent by pharmacies within the ED to deliver the medication required to treat the patients. The fifth sub-factor “*Transportation effectiveness*” determines whether the hospital has the minimum number of ambulances required by the government regulations to provide a timely emergency care service to users. It is also of interest to consider the role of the sterilization process, more important in pandemic times. In this respect, the inclusion of this decision element will allow ED managers to know if the ED has properly implemented the disinfection and sterilization processes requested to destroy pathogenic and non-pathogenic microorganisms with a high risk of damage and spread within the ED wards. Another aspect to include in this hierarchy is the “*Efficacy of non-core activities*”, which assesses how well the maintenance, cafeteria, laundry, and surveillance departments support the ED operations.

The performance of healthcare operations is also affected by the “Human talent (H5)” involved in each service unit [69]. In this case, this dimension has been represented by four sub-criteria. The two first pillars are the “*Number of specialists*” and *“Number of general practitioners”,* which evaluate whether the number of available specialists/general physicians is enough to respond to the ED demand during a COVID-19 peak. In parallel, “*Certification in Advanced Life Support*” denotes the proportion of medical staff trained in Advanced Life Support, which is highly required in the presence of a rapidly evolving virus. Ultimately, “*Number of nurses*” evaluates the balance between the number of nurses assisting in the ED against the demand in the peak periods of the COVID-19 pandemic.

In the meantime, logistic managers are interested in constantly monitoring the supply chain performance underpinning the ED operations during this outbreak, especially those related to the medication and accessories needed in pandemic environments and seasonal respiratory epidemics (H6). In particular, four sub-categories are taken into account; “*Readiness of accessories and instrumentation*” allows decision-makers to know the inventory fill rate associated with the instruments and medical accessories (e.g., high-flow cannulas) required by the medical staff to provide an effective treatment for COVID-19 patients. The decision-makers also have to carefully examine the “*Supplies fill rate”* to ensure the timely and correct provision of cover masks, oxygen, medical gloves, face shields, goggles, medical gowns, and aprons as recommended by the WHO [70], especially for patients and medical staff in the frontline. Further, “*Medication fill rate*” is considered given the necessity for ensuring the medication inventory levels required to assist the forecasted number of incoming COVID-19 patients. Finally, the “*Bed occupancy rate*” verifies whether the current ED bed configuration is sufficient to address the expected demand due to COVID-19 [71].

On a different tack, disaster managers are called to constantly evaluate the *“Quality in healthcare (H7)”* provided to COVID-19 patients, aiming to diminish the risk of mortality, potential COVID-19 infection sequelae, and cost overruns. In this regard, five aspects have been included to offer a wide overview of this dimension: “*Mean physician waiting time*”, “*Patient satisfaction”, “Mean length of stay”,* “*Re-entry rate*”, and “*Waiting time for triage categorization*”. The “*Mean physician waiting time*” element measures the average waiting time experienced by COVID-19 patients before being treated in the ED. The second sub-criterion allows us to determine the patient’s perception regarding the ED care received during the pandemic, so that specific process inefficiency drivers can be further identified and tackled. In parallel, healthcare decision-makers are advised to monitor the “*Mean length of stay*” for lessening the exposition time to in-hospital microorganisms that may worsen the health status of admitted COVID-19 patients as well as to reduce the associated ED overcrowding [72]. The “*Re-entry rate*” is another performance indicator to be analyzed by hospital administrators since it is linked to the effectiveness of diagnosis, treatments, and post-discharge control during the COVID-19 pandemic. Likewise, continuously estimating the “*Waiting time for triage categorization*” will allow ED administrators to define how rapidly the triage configuration is responding to the admission rate in the presence of a fast-evolving virus [73].

Finally, this study included the “*Patient safety*” factor, an emerging healthcare dimension with increasing attention paid by the authorities, taking into account the reports evidencing gaps between the protocols and daily medical practice [74]. In this respect, four related aspects have arisen: (i) “*Hospital-obtained infections*” have caused the death of thousands of people per year worldwide, and these figures could augment during the COVID-19 outbreak if suitable measures are not taken timely within the ED wards. Another source of patient safety problems is associated with the (ii) “*Medication mistakes*”, which could trigger worsening health conditions for the COVID-19 patient. A third aspect of interest in this cluster is the (iii) “*Clinical diagnosis mistakes*”, which could represent a late medical intervention or an incorrect allocation of scarce resources. Ultimately, ED managers need to control the (iv) “*Patient identification errors*”, which may ramp up given the large volume of ED admissions expected in each COVID-19 wave. In this case, such mistakes could lead to incorrect treatment plans and medications endangering patients’ life.

### 4.3. Estimation of Intuitionistic Fuzzy Weights: The IF-AHP Method

This section presents how the IF-AHP method was applied to obtain the relative weights of criteria and sub-criteria concerning the ED performance during the COVID-19 outbreak. First, a questionnaire-based tool was designed to collect the paired judgments emanated from the experts according to phase 2 (see Section 3.1). Then, the degree of knowledge of the experts was determined using the scale described by Boran et al. [51] and in Equation (6) (Table 1). In this case, the decision-makers with higher levels of experience were *DM1, DM2, DM3, DM4*, and *DM5* (w=0.1714), given their ample knowledge and expertise in the operability of emergency departments during pandemic situations. Afterward, the pairwise comparisons of the experts were aggregated via the IFWA operator (Equations (7) and (8)). An example of this step (matrix *“Healthcare equipment—sub-criteria”*) is described in Table 2. Table 3 shows the normalized priorities of the *Healthcare equipment* sub-factors. The resulting local weights (LW), overall weights (OW), and CRs are enlisted in Table 4.

**Figure 4:**
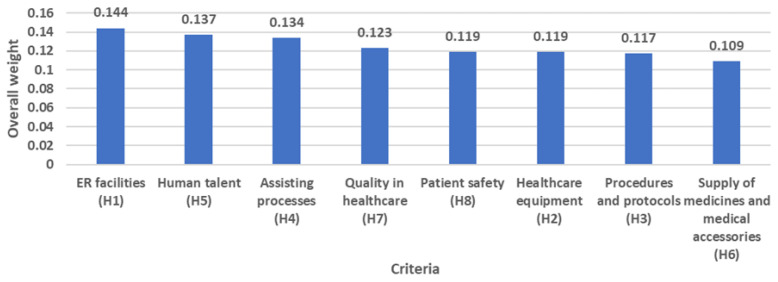
Ranking of criteria in the ED performance evaluation model.

**Figure 5:**
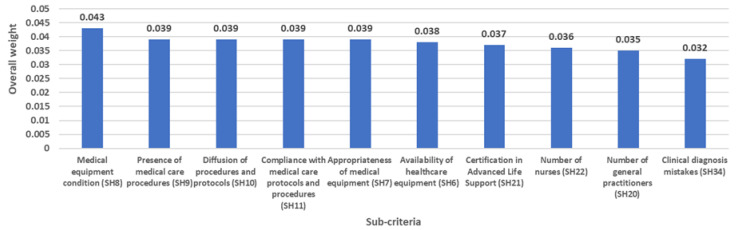
Ranking of sub-criteria in the ED performance evaluation model.

The ranking of criteria and top-ten classification of sub-criteria are presented in Figure 4 and Figure 5. In this regard, the IF-AHP outcomes revealed that the most significant decision elements in assessing the emergency department’s performance during the COVID-19 Outbreak were “ER facilities” (OW = 0.144), “Human talent” (OW = 0.137), and “Assisting processes” (OW = 0.134). However, there were no significant differences concerning the importance of the factors included in the performance evaluation model; in fact, the gap between “ER facilities” (first) and “Supply of medicines and medical accessories” (eighth) is only 0.035. This can be explained considering the multidimensional nature of emergency departments [75], the complexity of the social scenario imposed by the COVID-19 pandemic [76], as well as changes in the legal regulations and health guidelines designed to promptly respond to the new ED dynamics. Therefore, EDs must create a set of multifactorial strategies including all the criteria to increase their preparedness level against the current outbreak but also in the face of future pandemic events.

It is also essential to delve into the reasons justifying the importance of the first three criteria. Concerning “*ER facilities*” (OW = 0.144), ED managers have been challenged to tackle the overcrowding problem, especially during the pandemic peaks, where emergency wards are forced to adapt the existing infrastructure to meet the growing volume of COVID-19 admissions [77]. Different studies highlighted the importance of addressing the design of hospitals in the face of future pandemics [78,79]. This is pivotal considering the importance of ensuring the person-to-person distance to minimize the COVID-19 infection rates within the EDs. On the other hand, the second place of “*Human talent*” (OW = 0.137) in the overall ED performance is justified by the necessity of acquiring competencies suitably responding to the pandemic complexity while ensuring the sufficient workforce volume to address both urgent and non-urgent COVID-19 admissions. Explicitly, the ED medical staff has become one of the main restrictions experienced by EDs, especially regarding the availability of distinct specialists required to provide complete emergency care. Besides, gaps in workforce adaptions and practices directly diminish the effectiveness of crisis management plans and the subsequent overall ED response to the pandemic, which ultimately compromises patient satisfaction, door-to-doctor waiting times, and financial sustainability. Likewise, it is of paramount importance to guarantee the effective coordination, integration, and interaction among the “*Assisting processes*” (OW = 0.134) pillaring the emergency care services. Mismatches in the health supply chains may signify a delayed diagnosis and treatment for a COVID-patient, thereby ramping up the mortality likelihood and the use of more complex services (i.e., hospitalization, surgery, intensive care) for addressing the complications derived from the virus progress.

It is also fundamental to analyze the top-ranked sub-criteria to specifically create and unfold rigorous multidimensional strategies scaling up the ED performance during the pandemic peaks. *Medical equipment condition* (OW = 0.043) was highlighted as the most prominent aspect to monitor during each COVID-19 wave. The pandemic imposes new technical, quality, and sanitary guidelines to efficaciously satisfy the needs of COVID-19 patients as established by Garzotto et al. [80]. Not being prepared with the appropriate technology may represent delays in the provision of supplemental oxygen therapy, mechanical ventilation, and drugs often necessitated in these patients. Medical equipment management is therefore vital to alleviate the demand pressure and becomes a constant challenge for policy-makers in the presence of resource allocation constraints. However, the outbreak complexity also demands *appropriate medical equipment* (OW = 0.039), in other words, the devices with the pertinent operational characteristics in a pandemic context of respiratory disease. In this respect, non-adherence to the COVID-19 clinical care management guidelines and interventions may lead to an incorrect definition of the medical devices required in each scenario and subsequent poor support for protection, triage, diagnostic, and clinical care processes. Likewise, the *presence of medical care procedures* (OW = 0.039), *diffusion of procedures and protocols* (OW = 0.039), and *compliance with medical care protocols and procedures* (OW = 0.039) have gained increasing relevance for ED administrators when seeking optimal operational performance during this crisis. Undoubtedly, the COVID-19 pandemic has generated changes in health systems worldwide, and the protocols for treating this virus and other pathologies in this context have not been the exception. Since the beginning of the pandemic, the World Health Organization and other international/national public health organizations have designed and recommended the implementation of biosafety protocols for health workers as well as service providers, patients, and their families. However, deficiencies are evident regarding the availability of safety protocols and diagnostic/therapeutic protocols to care for suspected and confirmed COVID-19 patients [81] or to avoid the risk of COVID-19 infection when managing other pathologies. Similarly, in a study by Eftekhar Ardebili et al. [82], one of the most significant difficulties detected was the frequent change in protocols, prevention, and treatment methods. The *availability of healthcare equipment* (OW = 0.038) is another challenging aspect given the numerous reports on the scarcity of these devices during the most demanding times of the pandemic [80,83,84]. A low inventory of these medical devices could lead to operating below normal standards of care, which reduces the support offered by the EDs during COVID-19 surges. Likewise, *“Certification in Advanced Life Support”* (OW = 0.037) deserves special attention due to the shortage of professionals with solid theoretical knowledge and practical experience in managing cardiopulmonary or cardiovascular emergencies, a situation that has worsened within the COVID-19 context given their direct consequences in both respiratory and circulatory systems [85,86]. In addition, based on the scientific evidence, it has been identified that poor preparation for ED care and low funding for staff training inhibits the improvement and development of emergency health capacities [87]. Other sub-factors to carefully monitor are related to the availability of medical personnel: “*Number of nurses*” (OW = 0.036) and “*Number of general practitioners*” (OW = 0.035). Shortage of medical staff is associated with a lower quality of care [88] and becomes a driver of poor health outcomes in infected patients. As the virus drives up the cases and the ensuing ED admissions, long-standing difficulties including worker burnout and turnover have worsened, thereby limiting the number of available nurses and physicians on the frontline.

A consistency evaluation was also carried out for the aggregated intuitionistic fuzzy decision matrixes based on Equation (9). In this case, all matrixes were found to be consistent (CR < 0.1) (Table 4), which evidences the high quality of the decision-making process geared towards the estimation of criteria and sub-criteria relative priorities. Therefore, the resulting weights can be employed for supporting the CoCoSo application, the subsequent ED ranking, and the identification of the interventions that need to be delineated for enhancing the preparedness and response of these units against the current outbreak. The biases were also minimized thanks to the correct selection of decision-makers, accompanied by training and support during the comparison process. This is also proved through the consistency results achieved in large-sized matrices (*n* > 5) where most discrepancies occur: criteria (CR = 0.058; 8 elements) and H4 sub-factors (CR = 0.046; 7 elements). Finally, it is important to note that some criteria and sub-criteria weights can be used in similar ED performance evaluation processes considering pandemic events and/or seasonal respiratory diseases.

### 4.4. Intuitionistic Interdependence and Feedback: The IF-DEMATEL Approach

IF-DEMATEL was later applied to analyze the interdependence and feedback among performance criteria/sub-criteria. A data-collection instrument was also utilized to gather the paired judgments supporting the assessment of interrelationships within the proposed MCDM model. The decision-makers used the evaluation scale stated in Section 3.2. Training and close follow-up were provided by leading researchers to minimize the inconsistencies in the evaluation process. As a result, an initial intuitionistic fuzzy matrix $\tilde{Z_{k}}$ containing the comparisons per each cluster-expert was achieved. Table 5 shows an example of this step using Expert 1 insights concerning the interdependence among human talent sub-factors. Then, these matrixes are defuzzified according to the two-stage approach outlined in Section 3.2. In this respect, the IFSs were first transformed into a standard fuzzy subset by employing the equation $\mu \left(x\right)=\frac{1}{2}\left(1+\mu_{I}\left(x\right)-v_{I}\left(x\right)\right)$ (see example in Table 6). Subsequently, a defuzzification function was applied to convert the ensuing subsets into a triangular fuzzy number (TFN) by using Equations (12) and (13). The resulting crisp direct-relation matrix $Z_{1}\;$for the human talent sub-criteria (Expert 1) is presented in Table 7. Following this, the aggregated initial direct-relation matrices Z are produced after applying Equation (14) (see example in Table 8). We also calculated the convergence index (Equation (15)) for each matrix as follows: criteria (0.05), ER facilities (0.023), healthcare equipment (0.006), procedures and protocols (0.004), assisting processes (0.05), human talent (0.01), supply of medicines and medical accessories (0.017), quality in healthcare (0.032), and patient safety (0.022). In this case, none of these matrixes were found to be inconsistent (≤0.05), and the IF-DEMATEL results can be therefore deemed as reliable for supporting the cause–effect analysis within the ED performance model. Afterward, the normalized direct relation matrix *N* was derived via Equations (16) and (17) (see Table 9). The total influence matrix *T* was then estimated by using Equation (18) (see Table 10). Finally, Table 11 depicts the *prominence* (D + R) and *relation* (D—R) scores calculated via Equations (19) and (20) to identify which criteria or sub-criteria can be classified into the cause (dispatcher) and effect (receiver) groups. Thereby, the health authorities can be informed on which elements should be intervened with for enhancing the ED response in new COVID-19 waves and similar seasonal respiratory diseases.

**Figure 6:**
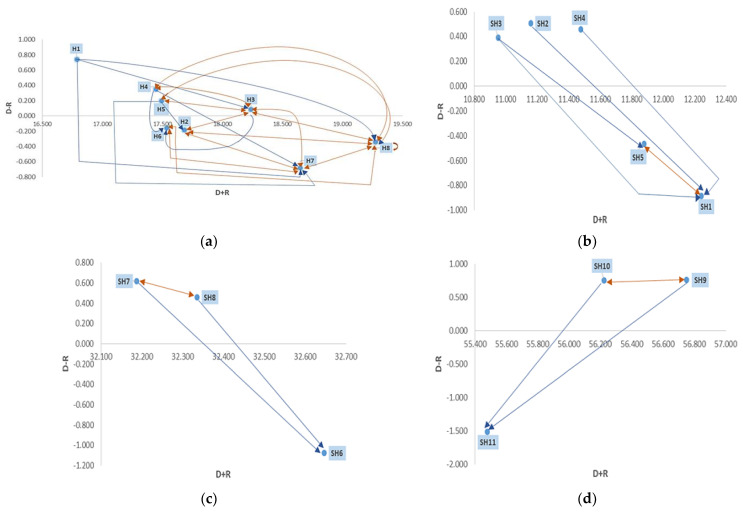

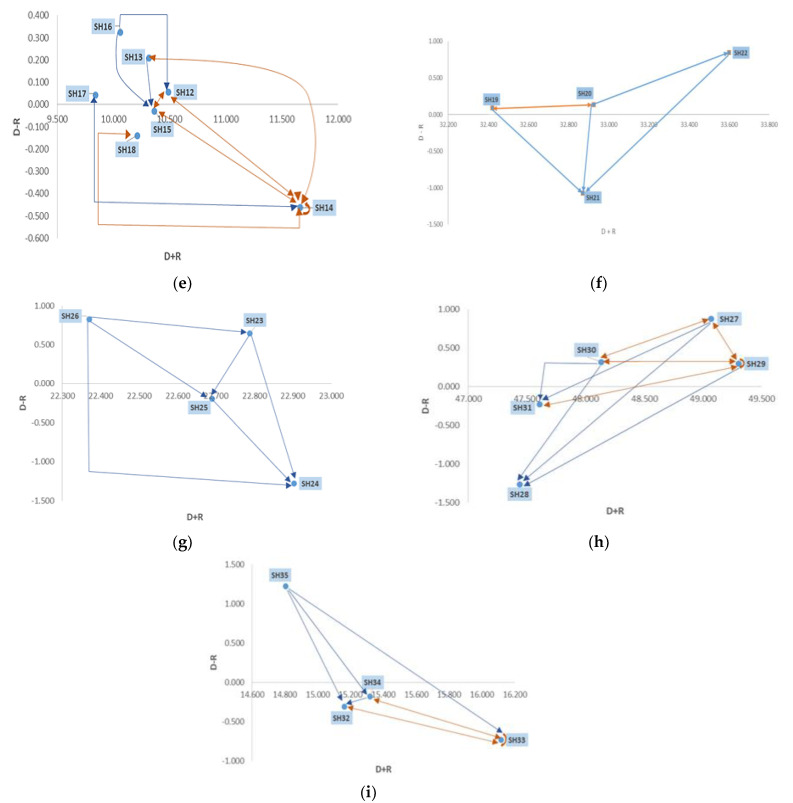
Influential-relation map for (**a**) criteria, (**b**) ER facilities, (**c**) healthcare equipment, (**d**) procedures and protocols (**e**) assisting processes, (**f**) human talent, (**g**) supply of medicines and medical accessories, (**h**) quality in healthcare, and (**i**) patient safety.

Finally, the threshold value *θ* was obtained per each matrix to derive the impact-digraph map (IRM) and consequently identify the significant interrelations among criteria and sub-criteria (see Figure 6a–i). For example, Figure 6a illustrates the cause–effect interrelations among criteria. In this case, the reference value was set as $\theta =\frac{71.557}{8^{2}}=1.118$. The results endorse the dispatching roles of *ER facilities (H1), Medical guidelines (H3), Assisting processes (H4),* and *Human Talent (H5)* whilst underpinning the effect nature of *Healthcare equipment (H2), Supply of medicines and medical accessories (H6), Quality in healthcare (H7),* and *Patient safety (H8).* The ED administrators, sector managers, and health authorities then need to envisage intervention strategies focused on these factors to guarantee the adequate provision of emergency services during the pandemic. This is explicated by the fact that different healthcare organisms have recommended modifying structural aspects of H1, H3, H4, and H5 to efficiently respond to the pressure imposed by this outbreak. Besides, these factors compound the operational ED core, which implies that plans on H2, H6, H7, and H8 depend on what is defined by the dispatchers. It also became glaring that *Procedures and Protocols* has the highest prominence value (D + R = 18.239) among the dispatchers, thus being the most influential factor in ED performance during the COVID-19 outbreak. Since the pandemic onset, health services around the world have had to make adjustments to their procedures, protocols, and work guidelines to incorporate advances in triage, management, and treatment of the disease as well as other pathologies to avoid intra-hospital infections and accelerate the emergency care provision. This finding is consistent with that of Shrestha et al. [89], who highlighted that EDs are generally prepared to deal with mass casualties secondary to traffic accidents but lack preparation and experience for biological-type incidents. Hence, the rapid and efficient implementation of protocols is required to address the changing nature of the pandemic. Particular attention should be also paid to the high volume of bidirectional relationships (H4–H8, H5–H8, H7–H8, H2–H8, H3–H8, H6–H8, H6–H7, H2–H7, H3–H7, H2–H3, H3–H4, and H3–H5) within the ED performance model (see the orange arrows). Specifically, *Patient safety* (H8) was found to be the element with the major number of these interactions, thereby making it an important improvement point to consider in the contingency plans. For example, the interaction H5–H8 is justified by the fact that deficiencies in support programs for health personnel during the COVID-19 pandemic could potentially restrict organizational resilience and negatively affect patient safety [89]. Therefore, EDs must include programs not only directed towards the hard competencies but also those considering the emotional support needed by the staff to work in a safe, resilient, and flexible manner.

In addition to analyzing the interactions among criteria, it is essential to detail the interdependence within the clusters. The interrelations for the *ER facilities* dimension have been graphed in Figure 6b. In this dimension, the threshold value was defined as $\theta =\frac{24.591}{5^{2}}=0.984$, thereby allowing us to state that *Physical status (SH1)* and *Bed availability (SH5)* are receivers while *Airing and lighting (SH3)*, *Sanitary installations (SH4)*, and *Delineation of emergency areas (SH5)* are ED performance drivers. Different studies have reported changes in the use of emergency medical facilities due to the COVID-19 pandemic [90,91]. Specifically, EDs have adapted their wards to attend to the growing demand of infected patients, which added to the regular volume of admissions related to other pathologies. In this sense, *Airing and lighting (SH2)*, *Sanitary installations (SH3)*, and *Delineation of emergency areas (SH4)* are essential to guarantee adequate, safe, and comfortable physical spaces for COVID-19 patients and their families. These factors must be taken into account when planning changes and expansions in the ED infrastructure, so that physical, sanitary, and comfort conditions can be ensured under the COVID-19 management guidelines. For instance, Łukasik and Porębska [77] proposed the conversion of one or more wards into pavilions for COVID-19 patients. Further, the use of simulation tools can provide different layout alternatives that allow ED administrators to plan the ED infrastructure against new COVID-19 waves and respiratory seasonal diseases. On the other hand, there is a double-sided arrow in between the receivers, which denotes that the installation of hospital beds must be accompanied by the adaptation of the ED physical status to provide effective care.

An influential-relation graph has been also outlined to reveal the inner effects among *Healthcare equipment* sub-criteria (Figure 6c). A $t_{ij}>\theta \;||\theta =\frac{48.583}{3^{2}}=5.398$ denoted the significant interdependencies in this cluster. In summary, the outcomes revealed that *Availability of healthcare equipment (SH6)* was identified as the only receiver whilst *Appropriateness of medical equipment (SH7)* and *Medical equipment condition (SH8)* were classified as dispatchers. Additionally, a feedback relationship between SH7 and SH8 was evidenced. It is also important to point out that SH8 was found both as the most critical sub-criterion (LW = 35.8%) and the most influential element (D + R = 32.335) in this cluster. Consequently, ED managers, healthcare cluster directors, Ministries of Health, Procurement departments, and regulatory agencies are advised to implement the following recommendations [92]:

- ensure medical equipment consumables for a minimum 3 months;
- provide training on the use of vital medical devices for physicians and nurses;
- adapt the medical equipment to the healthcare workforce and infrastructure available in the ED;
- design specialized maintenance plans to further enhance the functionality of critical devices during the pandemic;
- test the conformity of the device with the minimum requirements established by the WHO.

A threshold value $\theta =\frac{84.225}{3^{2}}=9.358$ was defined to detect significant interrelations in the *Procedures and protocols* cluster. The impact digraph with this information is shown in Figure 6d. The graph uncovers that the *Presence of medical care procedures (SH9)* and *Diffusion of procedures and protocols (SH10)* are the main performance drivers in the H3 criterion, whereas *Compliance with medical care protocols and procedures (SH11)* was identified as the effect. It is also good to mention that the *Presence of medical care procedures* is the sub-criterion with the highest prominence value (D + R = 56.750). This finding confirms what was stated by Tanja et al. [93] regarding the need to establish clinical practice guidelines and recommendations to support therapeutic decisions considering the challenges imposed by the COVID-19 pandemic. In addition, there is a two-way relationship between SH9 and SH10 (D + R > 50). This means that an effective diffusion of these protocols partially depends upon whether the ED has incorporated these guidelines in its quality management system. Thereby, the ED can grant a long-term framework for planning, execution, verification, and improvement, considering the risk of future pandemic events. Not less important was the low adherence to COVID-19 procedures reported in some EDs and which was associated with poor dissemination and structuring problems in these protocols [94].

Interrelations within the *Assisting processes* were also mapped to identify intervention priority areas (Figure 6e). In this instance, the significance limit was calculated to be $\theta =\frac{36.476}{7^{2}}=0.744$. As a result, *Efficacy of radiology process (SH12)*, *Efficacy of clinical lab (SH13), Transportation efficacy (SH16),* and *Efficacy of sterilization process (SH17)* were categorized as dispatchers, whilst *Efficacy of hospitalization process (SH14)*, *Efficacy of pharmaceutical service (SH15)*, and *Efficacy of non-core activities (SH18)* were found to be receivers. In the cause group, *Efficacy of radiology process (SH12)* has the highest D + R value (10.489) and is then considered the most influencing subfactor when evaluating the assisting processes. This finding is supported by Santos et al. [95], who reported that workflows in radiology processes were inefficient when addressing ED patients with COVID-19 pneumonia, thereby producing increased lengths of stay and waiting times. On a different tack, the feedback relationship SH12–SH13 deserves special attention and encourages healthcare managers to adopt strategies reducing turnaround times and augmenting reliability in radiology and clinical laboratory processes. This is essential to ensure rapid medication administration depending on the radiology and laboratory results. On a different note, EDs are advised to have an optimal number of ambulances to provide emergency services to COVID-19 patients at a reasonable time. Complementary to these proposals, effective disinfection and sterilization processes of ED wards are necessitated to avoid intra-hospital infections and the ensuing health complications in those patients with baseline diseases.

The IF-DEMATEL outcomes additionally uncover the interrelations among *Human talent* sub-criteria. In this cluster, a reference value $\theta =\frac{65.908}{4^{2}}=4.119\;$ (Figure 6f) was defined by the decision-making group to identify the significant interactions. Consequently, *Number of specialists (SH19), Number of general practitioners (SH20),* and *Number of nurses (SH22)* were recognized as dispatchers, whereas *Certification in Advanced life Support (SH21)* was classified as a receiver. Likewise, *Number of general practitioners* was concluded to be the most influential sub-factor (D + R = 33.599). In this regard, Long et al. [85] and Cao et al. [86] declared that having essential medical personnel for COVID-19 patient management in emergency units helps to balance the response of the healthcare system with the demand, especially in epidemiological peaks where the available staff is subject to stress. It is thus imperative that ED administrators implement strategies focused on the provision of human talent according to demand, suitable personnel scheduling, and constant review of working/biosafety conditions. In addition to these considerations, the feedback interrelation between SH19 and SH20 demands support from health information technologies optimizing the workflows among physicians with different specialization levels during the pandemic. In this way, in-time and suitable diagnosis and treatment can take place as needed in the presence of a rapidly evolving virus.

Concerning the *Supply of medicines and medical accessories* cluster, the adopted threshold value was $\theta =\frac{45.375}{4^{2}}=2.836\;$. Based on this insight, the impact-relation map (Figure 6g) was found to reveal the dispatching nature of *Readiness of accessories and instrumentation (SH23)* and *Bed occupancy rate (SH26),* while *Supplies fill rate (SH24)* and *Medication fill rate (SH25)* were categorized as receivers. In this respect, it is vital to permanently monitor the availability of beds, consumables, and accessories to underpin planning and management systems that raise alarms in response to high stockout probability. These systems can support input requirements, purchasing processes, and inventory models guaranteeing satisfactory fill rates whilst complying with quality and biosafety criteria. Such strategies are called to tackle the potential adverse events related to inadequate resource management during this global crisis [96].

An impact diagram was also established for the *Quality in healthcare* sub-factors (Figure 6h). The estimated reference value was set as $\theta =\frac{120.775}{5^{2}}=4.831$. In this case, *Mean physician waiting time* (SH27), *Mean length of stay* (SH29), and *Re-entry rate* (SH30) were classified into the cause group, whereas *Patient satisfaction (SH28)* and *Waiting time for triage categorization (SH31)* were included in the effect group. Likewise, it was found that SH29 has a strong effect on the receivers (D + R = 49.301). In addition, the high prominence values (D + R > 40) show the existence of strong correlations among these sub-criteria. Considering the above, it is necessary to foster the use of simulation and lean six sigma methodologies to evaluate whether the current installed ED capacity is sufficient to satisfy the maximum waiting times and lengths of stay established by the regulatory agencies. It will be thus possible to pinpoint non-added-value activities that need to be removed through improvement operational strategies. If these dispatchers are intervened, the impact is significant on the emergency care quality provided to COVID-19 patients during this period. Therefore, interventions aimed at reducing quality errors can address low satisfaction levels and generate a useful body of knowledge for managing patient perception in future pandemics [97].

Ultimately, a digraph map was generated for the *Patient safety* cluster (Figure 6i). The threshold value was defined to be $\theta =\frac{30.701}{4^{2}}=1.919$ by the expert team. In this dimension, *Patient identification errors (SH35)* was detected to be the only dispatching sub-factor. On the other hand, *Hospital-obtained infections (SH32)*, *Medication mistakes (SH33)*, and *Clinical diagnosis mistakes (SH34)* were concluded to be receivers. The map also reveals feedback interrelations (SH32–SH33 and SH33–SH34) that should be analyzed by the decision-makers to propel better patient safety practices. ED managers are expected to implement comprehensive strategies for reducing patient identification errors and avoid more severe health complications and the use of upstream services accordingly. Incorrect patient data may lead to errors in medical diagnoses and medicine administration. Some strategies may include the implementation of central software supported by QR codes or electronic identifiers storing the patient data along the ED pathway. Moreover, it will be necessary to design patient data registration protocols underpinning the correct deployment of these technological solutions in the wild while performing audits evidencing the gaps in practice.

### 4.5. Calculation of ED Performance Index: The CoCoSo Method

The final step of this approach involves applying the CoCoSo method to fundamentally pursue three objectives:

- calculate the ED performance index (EDPI) to rank the Turkish emergency centers (D1, D2, and D3);
- detect weaknesses in each ED to improve their overall response against pandemic scenarios caused by COVID-19 and similar respiratory diseases;
- establish individual improvement plans supporting interventions by ED administrators and healthcare authorities.

The CoCoSo implementation was initiated by defining a key performance index (KPI) per each sub-criterion. The list of KPIs and their mathematical formulas are shown in Table 12. The metrics were determined considering the healthcare regulations, pertinent scientific literature, and balanced scorecards established in the participant institutions. Each indicator was measured in each ED to construct the initial decision matrix *M* (Table 13). Table 13, derived from Equation (21), also compiles the overall sub-criteria weights emanating from the IF-AHP method, thereby tackling the weighting procedure weakness detected in most of the outranking techniques. Following this, the decision matrix *M* was normalized using Equations (22) and (23). As a next step, we estimated the sum of weighted comparability $\left(S_{i}\right)$ and the power-weighted comparability sequences $\left(P_{i}\right)$ for each ED using Equations (24) and (25) (see Table 14). Then, the three aggregated appraisal scores were computed $\left(M_{ia},\;M_{ib},M_{ic}\right)$ via Equations (26)–(28), considering λ = 0.5 (see Table 14). Finally, the aggregated $M_{i}$ score, now called EDPI (Equation (29)), was calculated for each emergency center (see Table 14).

An essential element in analyzing the CoCoSo method results is the ED performance index (EDPI). In this case, the highest EDPI that an emergency department can reach was 3.359, which was the score achieved when the sub-criteria values are equal to the ideal response scenario. Therefore, if interventions are not implemented to tackle the deficient performance factors, it is expected that the EDPI will tend to decrease over time, with negative effects for both the emergency centers (i.e., adverse events, cost overruns, complaints, legal sanctions) as well as for patients (long-term sequelae, higher mortality risk due to COVID-19) and medical personnel (stress, work overload, risk of contagion). The results show significant gaps between the ED response against the pandemic and the desired performance (maximum EDPI), which is proven through a mean difference of -1.225 with a standard deviation of 0.3267. Regarding the ranking, D2 (*M*i = 2.401; *M*2/Máx EDPI = 70.57%) turned out to be the emergency department with the best performance followed by D1 (*M*i = 2.243; *M*2/Máx EDPI = 67.57%), while D3 was concluded to be the one with the lowest performance (*M*i = 1.740; *M*2/Max EDPI = 52.42%). It is evident that there is much room for improvement in the Turkish EDs whose performance did not overpass 75% in any of the cases.

Given the above-mentioned, specific intervention plans are required to improve the overall response of these emergency departments against the pandemic. The weaknesses are those sub-criteria with normalized values equal to or close to 0. For example, D1 presented the lowest % of ED wards with suitable infrastructure status (80%), which is consistent with the findings detected by Winkelmann et al. [98] and Walton et al. [99]. They specifically highlighted the limitations created by the physical status of EDs, which trigger inadequate isolation zones, triage wards, and testing facilities, thereby restricting the COVID-19 patient flow within the ED. To overcome this unprecedented challenge, it is advised to:

- repurpose non-health facilities inside (i.e., administrative offices) and outside (i.e., hotels, parking lots) the EDs to accommodate patients with mild COVID-19 and low risk of severe complications;
- implement infrastructure adequation plans to rapidly set up ED areas with poor infrastructure condition;
- transfer low-risk COVID-19 patients to other regions with spare capacity.

On the other hand, D1 reported the lowest bed availability (2200 beds) and is therefore an improvement area for this unit. In this regard, Remuzzi and Remuzzi [100] stated that bed capacity plays an essential role in managing the increasing volume of COVID-19 presentations in the ED. In this sense, it is recommended that those responsible for the emergency centers to:

- design predictive models estimating the number of EDs required depending on social factors, the virus spread, and policies implemented by the government;
- establish collaborative agreements with the bed supply chain actors where their production and logistics capabilities can be mostly destinated to the EDs with bed shortages;
- develop MCDM models identifying which COVID-19 patient can be safely discharged home and who should be immediately attended to, supporting scarce resource allocation activities and minimizing the mortality rate.

A grave concern in this analysis is that none of the showcased EDs evidenced continuous monitoring of adverse events related to COVID-19 patients. This may be explained by the lack of sufficient healthcare quality professionals who were mostly focused on monitoring the timeliness and efficacy of the care provided to this population. Besides, the pandemic placed enormous pressure on the healthcare system and especially the EDs as the primary gate to the entire hospital structure, which led ED administrators to prioritize activities including resource management and the implementation of safety measures for the frontline medical staff. This finding was also declared by Sharp et al. [101], who additionally expressed that this task was impacted by the fear that physicians had of contracting COVID-19, the restricted volume of protective equipment, and the patient flow variations. In this respect, EDs are invited to:

- develop practical artificial intelligence models predicting the occurrence of adverse events in COVID-19 patients;
- assign staff from the quality departments to constantly control and investigate the adverse events related to this disease;
- create resilience plans discriminating which activities should be rolled out by the ED depending on the risk of adverse events.

Likewise, implementing interventions to reduce the average waiting time for radiology results deserves special attention considering that D1 and D3 obtained times above the ideal scenario (>60 min), which is consistent with the findings outlined by Santos et al. [95] regarding the long waiting times in radiology units due to demand surges and the inefficient inflows of traditional care. It is also important to note that the *Efficacy of the radiology process* was identified as the most influential sub-factor in the performance model. Therefore, interventions focused on improving patient care flows are widely necessitated. For instance, Lean Six Sigma techniques can provide a way to reduce the turnaround time by removing non-value-added activities. Another recommended intervention is the use of information systems facilitating physicians’ access to radiology results. In addition, efforts should be also concentrated on the development of artificial intelligence models with high accuracy in predicting the X-ray outcomes based on easy-to-collect predictors. On a different note, one of the major shortcomings detected in D2 was the *Efficacy of pharmaceutical service,* where the average lead time for medication delivery was estimated to be 1 h. Medication shortage increases the risk of mortality in COVID-19 patients, who may need sedatives in case of intubation/mechanical ventilation and antimicrobials for therapy [102]. Some strategies may be implemented to address this problem:

- create inventory models with high fill rates responding to the COVID-19 dynamic;
- diminish the importation approval time to 24 h as some medication ingredients are mostly imported;
- augment the domestic installed production capacity of the medicines with shortages supported by demand prediction models.

Another conclusion related to this dimension is the non-implementation of *non-core activities* (maintenance, cooking, laundry, and surveillance), which were largely restricted during COVID-19, but they are fundamental in improving patient outcomes, controlling inflow, increasing availability of healthcare equipment, and improving adherence to sanitation standards. Procedures and protocols must be then designed in conjunction with the WHO to secure the normal functioning of these processes even in a devastating biological disaster such as the one experienced with COVID-19.

Other remarkable discoveries of this study are associated with the *Human talent* domain. CoCoSo revealed that D1 and D3 had a deficit in the number of specialists (oscillating between six and eight) and general practitioners (30 and 36, respectively), while D1 and D2 required a substantial number of nurses (ranging from 23 to 26). Moreover, D1 presented the lowest percentage of medical staff certified in Advanced Life Support (60%). The preceding is consistent with findings of Leite [103], who considers health personnel as a critical resource in managing this outbreak. However, there is a staffing shortage due to burnout and departures motivated by the two grueling years of experiencing a high level of stress. The initial installed capacity was insufficient compared to the one required during the COVID-19 peaks. To combat this problem, it is suggested to:

- tout retention bonuses for healthcare workers on the frontline of this pandemic;
- identify barriers to lessening medicine and nursing graduation rates in the universities to design collaborative interventions;
- train non-ALS-certified medical staff through courses designed by education authorities in conjunction with the Ministry of Health;
- assign specialists that were put on hold to stem the virus spread to assist physicians in caring for COVID-19 patients;
- form groups of physicians and nurses led by ICU clinicians to address patients with a higher risk of mortality;
- deploy virtual hospitals to identify COVID-19 patients with an urgent need for care and consequently diminish the patient flow arriving at the EDs.

When analyzing the *Supply of medicines and medical accessories* aspect, the primary concern is the *readiness of accessories and instrumentation* where D2 and D3 obtained the highest levels in the number of medical consumables and instruments needed to cover the current demand (85 and 100, respectively). This is partially explained by the reduced import from China [104] and the lack of accurate models predicting their demand. Some hospitals have reported a sharp rise in the daily consumption of masks and gloves [105]. Due to the preceding and given the criticality of this indicator, the recommendations include:

- establish agreements with the related supply chain actors for the purchase of medical consumables and instruments under the principles of quality, timeliness, flexibility, and scale economy;
- the use of information technologies for the efficient and reliable management of medical supplies;
- the development and implementation of protocols for the rational management of accessories and instrumentation;
- the design of inventory models responding to the changing dynamics of the pandemic.

Ultimately, a common deficiency linked to the overwhelming ED wards is the prolonged stays, which were estimated to be equal to and/or over 2.5 days in all the participant departments. The escalating demand depleted the hospitalization beds and ticked up the queue size in the EDs [106]. This is added to the rapid health worsening experienced by some COVID-19 patients who needed extra support from the medical staff and the application of respiratory therapies that may take more time than planned. The road ahead must include strategies providing short- and medium-term relief to the emergency care landscape. In summation, ED managers are expected to:

- implement machine learning models predicting the stay length of COVID-19 patients;
- optimally increase the number of hospitalization beds to decrease overcrowding and waiting times in the ED;
- identify the treatments that can be provided through homecare services so that the number of COVID-19 patients within the ED rooms can be substantially diminished;
- reduce the diagnosis times by lessening the radiology and lab turnaround times as further explicated above.

## 5. Conclusions

The COVID-19 pandemic has posed a significant burden to the EDs which has evidenced performance gaps that need to be narrowed through a collaborative network involving local/international regulatory agencies, health authorities, healthcare sector leaders, ED administrators, and hospital managers. In the meantime, designing multidimensional interventions is pivotal to upgrade the response of these units against new epidemiological waves and a forecasted sharp rise in the volume of ED presentations expected with seasonal respiratory diseases.

Despite extraordinary efforts made by the healthcare structure, COVID-19 is still with us and therefore imposes a major challenge for EDs, which requires them to constantly monitor their performance to avoid substantial affectations on the population’s health. Ensuring high standards in patient safety, medical equipment, human talent, ER facilities, procedures/protocols, assisting processes, supply of medicines/accessories, and quality will propel a satisfactory reply against similar scenarios. Even as the world enters a less acute phase of the pandemic, the healthcare spectrum dictates the appearance of new epidemics where ED will be again stretched. In this regard, hospital administrators are expected to sharpen disaster management plans and create a resilient operational structure capable of reacting effectively in the face of pronounced healthcare demands and a challenging social context.

Given the above, this paper has proposed the implementation of a hybrid intuitionistic fuzzy approach to assessing ED performance during the COVID-19 outbreak. A real case study comprising three Turkish EDs was employed to validate the suggested framework. The IF-AHP results revealed that the most critical factors in ED performance are “ER facilities” (OW = 0.144), “Human talent” (OW = 0.137), and “Assisting processes” (OW = 0.134). On the other hand, the IF-DEMATEL application uncovered that “Procedures and protocols” (D + R = 18.239) is the most influential aspect in the development of medium- and long-term disaster management plans for EDs. Lately, the CoCoSo outcomes showed that D2 (Mi = 2.401; M2/Máx EDPI = 70.57%) turned out to be the emergency department with the best performance. Nonetheless, interventions in ER infrastructure, human talent, assisting processes, healthcare quality, and supply chain resilience are recommended to enhance the overall response of the emergency care service provided by these institutions.

Our study can help government leaders and Ministries of Health to devise specific intervention points and therefore focused strategies where scarce resources can be better allocated, while diminishing the risk of long-term sequelae and mortality rate for COVID-19 patients. Likewise, it acts as a warning system that can also guide decision-makers in the timely deployment of social and political interventions. Not less important is the close follow-up offered by this approach regarding the effectiveness of rolled-out strategies, which is widely requested in healthcare environments with limited resources and no room for major investments.

The hybrid approach used in this work has provided very good results and is shown to be robust. However, some limitations should be commented on. First, there are other MCDM techniques that can be used to check the similarity of results as well as their quality, such as the case of the fuzzy-analytical network process. In this case, such a test was considered but was discarded because of the enormous work involved for the expert team in answering multiple different questionnaires adapted to the methodology of this technique. Second, the opinions of the expert team are always controversial, and although our work was made up of highly experienced people, we cannot be sure that a different group would choose the same criteria and sub-criteria or would have different evaluations that would lead to a slight variation in the results. Third, by using IF-AHP, we improve the weighting of the decision-makers in the expert team by considering their experience. Although the CR is used to detect discrepancies in the judgments of the experts, this only ensures the consistency of the expert team, not the quality of that team, i.e., if the team is made up of people with low experience; compliance with the minimum value of CR does not ensure good results of the analysis, only that the team is consistent. This reflection applies in the same way when proceeding with step 4 of IF-DEMATEL (appraisal of cause–effect interrelations among criteria and sub-criteria considering uncertainty). Fourth, when we are carrying out step 5 of the methodology (calculation of the ED performance index and ranking derivation) using the CoCoSo model, it is necessary to keep in mind that the initial choice of the KPIs for each sub-criterion is crucial for the subsequent ranking and design of improvement interventions. A sub-criterion can be measured in different ways (KPIs), so the choice of these is important as they can influence the results. In this sense, each indicator is measured in each ED to construct the initial decision matrix M, before compiling the overall sub-criteria weights.

For future work, we plan to extend this application to other international contexts where ED performance may be affected by other factors that have not been considered in this study. Besides, we aim to include fuzziness in the outranking technique to perform a comparative analysis based on the EDPI indicator. Complementary to these research lines, the EDPI is intended to be included as a technical measure in a discrete-event simulation model representing the operability of EDs during the COVID-19 pandemic. Thereby, improvement scenarios augmenting the EDPI can be prioritized by modelers and policymakers when deciding what solutions to implement in the context of the COVID-19 pandemic and similar social landscapes.

## Figures and Tables

**Table 1:** Relative priorities of decision-makers and their IFNs.

Decision-Maker	DM1	DM2	DM3	DM4	DM5	DM6
IFN	(0.9; 0.05; 0.05)	(0.9; 0.05; 0.05)	(0.9; 0.05; 0.05)	(0.9; 0.05; 0.05)	(0.9; 0.05; 0.05)	(0.75; 0.2; 0.05)
Weight	0.171429	0.171429	0.171429	0.171429	0.171429	0.142857

**Table 2:** Aggregated matrix—healthcare equipment sub-criteria.

	SH6	SH7	SH8
SH6	[0.020; 0.180; 0.800]	[0.105; 0.195; 0.698]	[0.123; 0.209; 0.666]
SH7	[0.132; 0.172; 0.695]	[0.020; 0.180; 0.800]	[0.100; 0.206; 0.692]
SH8	[0.174; 0.163; 0.662]	[0.114; 0.188; 0.697]	[0.020; 0.180; 0.800]

**Table 3:** Intuitionistic and normalized weights—healthcare equipment sub-criteria.

Sub-Criterion	Intuitionistic Fuzzy Weight	Non-Fuzzy Weight	Overall Weight
SH6	[0.083; 0.195; 0.722]	0.307	0.291
SH7	[0.084; 0.186; 0.729]	0.329	0.312
SH8	[0.103; 0.177; 0.720]	0.418	0.396
Total		1.054	1.000

**Table 4:** Local weights (LW), overall weights (OW), and consistency ratios (CR) in the ED performance evaluation model.

Criterion/Sub-Criterion	LW	OW	CR *
**ER facilities (H1)**		**0.144**	**0.046**
Physical status (SH1)	0.197	0.028	
Airing and lighting (SH2)	0.202	0.029	
Sanitary installation (SH3)	0.220	0.032	
Delineation of emergency areas (SH4)	0.179	0.026	
Bed availability (SH5)	0.201	0.029	
**Healthcare equipment (H2)**		**0.119**	**0.024**
Availability of healthcare equipment (SH6)	0.316	0.038	
Appropriateness of medical equipment (SH7)	0.326	0.039	
Medical equipment condition (SH8)	0.358	0.043	
**Procedures and protocols** **(H3)**		**0.117**	**0.003**
Presence of medical care procedures (SH9)	0.333	0.039	
Diffusion of procedures and protocols (SH10)	0.333	0.039	
Compliance with medical care protocols and procedures (SH11)	0.333	0.039	
**Assisting processes (H4)**		**0.134**	**0.046**
Efficacy of radiology process (SH12)	0.140	0.019	
Efficacy of clinical lab (SH13)	0.148	0.020	
Efficacy of hospitalization process (SH14)	0.121	0.016	
Efficacy of pharmaceutical service (SH15)	0.143	0.019	
Transportation efficacy (SH16)	0.140	0.019	
Efficacy of sterilization process (SH17)	0.141	0.019	
Efficacy of non-core activities (SH18)	0.168	0.022	
**Human talent (H5)**		**0.137**	**0.062**
Number of specialists (SH19)	0.214	0.029	
Number of general practitioners (SH20)	0.259	0.035	
Certification in Advanced Life Support (SH21)	0.267	0.037	
Number of nurses (SH22)	0.260	0.036	
**Supply of medicines and medical accessories (H6)**		**0.109**	**0.057**
Readiness of accessories and instrumentation (SH23)	0.228	0.025	
Supplies fill rate (SH24)	0.256	0.028	
Medication fill rate (SH25)	0.231	0.025	
Bed occupancy rate (SH26)	0.286	0.031	
**Quality in healthcare (H7)**		**0.123**	**0.097**
Mean physician waiting time (SH27)	0.198	0.024	
Patient satisfaction (SH28)	0.208	0.026	
Mean length of stay (SH29)	0.201	0.025	
Re-entry rate (SH30)	0.200	0.025	
Waiting time for triage categorization (SH31)	0.193	0.024	
**Patient safety (H8)**		**0.119**	**0.020**
Hospital-obtained infections (SH32)	0.239	0.028	
Medication mistakes (SH33)	0.244	0.029	
Clinical diagnosis mistakes (SH34)	0.266	0.032	
Patient identification errors (SH35)	0.251	0.030	

* The CR for the criteria matrix is 0.058.

**Table 5:** Initial intuitionistic fuzzy matrix—Expert 1 (human talent sub-criteria).

	SH19	SH20	SH21	SH22
SH19	[0; 0]	[0.9; 0.1]	[0.75; 0.2]	[0.9; 0.1]
SH20	[0.75; 0.2]	[0; 0]	[0.5; 0.45]	[0.75; 0.2]
SH21	[0.75; 0.2]	[0.9; 0.1]	[0; 0]	[0.5; 0.45]
SH22	[0.9; 0.1]	[0.5; 0.45]	[0.35; 0.6]	[0; 0]

**Table 6:** Initial intuitionistic fuzzy matrix in standard fuzzy subsets—Expert 1 (human talent sub-criteria).

	SH19	SH20	SH21	SH22
SH19	0.000	0.900	0.775	0.900
SH20	0.775	0.000	0.900	0.775
SH21	0.775	0.900	0.000	0.525
SH22	0.900	0.525	0.900	0.000

**Table 7:** Crisp direct-relation matrix $Z_{1}$—human talent sub-criteria.

	SH19	SH20	SH21	SH22
SH19	0.000	3.600	3.100	3.600
SH20	3.100	0.000	3.600	3.100
SH21	3.100	3.600	0.000	2.100
SH22	3.600	2.100	3.600	0.000

**Table 8:** Aggregated initial direct-relation matrices—human talent sub-criteria.

	SH19	SH20	SH21	SH22
SH19	0.000	3.017	2.850	2.933
SH20	3.183	0.000	3.350	2.767
SH21	2.683	2.833	0.000	2.850
SH22	2.850	2.850	2.917	0.000

**Table 9:** Normalized direct relation matrix—human talent sub-criteria.

	SH19	SH20	SH21	SH22
SH19	0.000	3.017	2.850	2.933
SH20	3.183	0.000	3.350	2.767
SH21	2.683	2.833	0.000	2.850
SH22	2.850	2.850	2.917	0.000

**Table 10:** Total influence matrix—human talent sub-criteria.

	SH19	SH20	SH21	SH22	D
SH19	0.000	0.324	0.306	0.315	16.532
SH20	0.342	0.000	0.360	0.297	17.222
SH21	0.289	0.305	0.000	0.306	15.898
SH22	0.306	0.306	0.314	0.000	16.256
R	16.394	16.377	16.973	16.164	

**Table 11:** Dispatchers and receivers in the ED performance evaluation model—COVID-19 context.

Code	Criterion/Sub-Criterion	D + R	D − R	Dispatcher	Receiver
H1	ER facilities	16.790	0.740	X	
SH1	Physical status	10.407	−0.721		X
SH2	Airing and lighting	9.528	0.408	X	
SH3	Sanitary installation	9.350	0.323	X	
SH4	Delineation of emergency areas	9.782	0.374	X	
SH5	Bed availability	10.115	−0.384		X
H2	Healthcare equipment	17.685	−0.183		X
SH6	Availability of healthcare equipment	32.646	−1.078		X
SH7	Appropriateness of medical equipment	32.187	0.618	X	
SH8	Medical equipment condition	32.335	0.459	X	
H3	Procedures and protocols	18.239	0.083	X	
SH9	Presence of medical care procedures	56.750	0.759	X	
SH10	Diffusion of procedures and protocols	56.223	0.754	X	
SH11	Compliance with medical care protocols and procedures	55.447	−1.513		X
H4	Assisting processes	17.445	0.349	X	
SH12	Efficacy of radiology process	10.489	0.056	X	
SH13	Efficacy of clinical lab	10.316	0.208	X	
SH14	Efficacy of hospitalization process	11.664	−0.463		X
SH15	Efficacy of pharmaceutical service	10.365	−0.030		X
SH16	Transportation efficacy	10.064	0.324	X	
SH17	Efficacy of sterilization process	9.842	0.044	X	
SH18	Efficacy of non-core activities	10.212	−0.139		X
H5	Human talent	17.494	0.195	X	
SH19	Number of specialists	32.926	0.138	X	
SH20	Number of general practitioners	33.599	0.844	X	
SH21	Certification in Advanced Life Support	32.871	−1.075		X
SH22	Number of nurses	32.420	0.093	X	
H6	Supply of medicines and medical accessories	17.536	−0.157		X
SH23	Readiness of accessories and instrumentation	22.788	0.649	X	
SH24	Supplies fill rate	22.903	−1.280		X
SH25	Medication fill rate	22.689	−0.196		X
SH26	Bed occupancy rate	22.370	0.828	X	
H7	Quality in healthcare	18.649	−0.683		X
SH27	Mean physician waiting time	49.070	0.879	X	
SH28	Patient satisfaction	47.439	−1.265		X
SH29	Mean length of stay	49.301	0.298	X	
SH30	Re-entry rate	48.133	0.320	X	
SH31	Waiting time for triage categorization	47.607	−0.232		X
H8	Patient safety	19.275	−0.343		X
SH32	Hospital-obtained infections	15.163	−0.313		X
SH33	Medication mistakes	16.116	−0.734		X
SH34	Clinical diagnosis mistakes	15.317	−0.182		X
SH35	Patient identification errors	14.805	1.229	X	

**Table 12:** Key performance metrics supporting the CoCoSo method.

Sub-Criterion	Performance Metric	Calculation Method
Physical status (SH1)	% of ED wards with suitable infrastructure status	$\frac{NEDWSI}{w}*100$ *NEDWSI*: Number of ED wards with suitable infrastructure *w*: Total number of ED wards
Airing and lighting (SH2)	% of ED wards without suitable illumination and cleansing	$\frac{NEDW-wSAL}{w}*100$ *NEDW*—*wSAL*: Number of ED wards without suitable illumination and cleansing conditions *w*: Total number of ED wards
Sanitary installations (SH3)	Availability of sanitary installations	If ready to use (1), otherwise (0)
Delineation of emergency areas (SH4)	Delineation of ED areas	If delineated (1), otherwise (0)
Bed availability (SH5)	Number of available beds in the ED	Number of beds available for COVID-19 patients in an emergency unit
Availability of healthcare equipment (SH6)	% of ready-to-use healthcare equipment	$\frac{NRTUE}{m}*100$ *NRTUE*: Number of ready-to-use healthcare equipment *m*: Total number of medical devices
Appropriateness of medical equipment (SH7)	% of medical equipment that is pertinent to COVID-19-related requirements	$\frac{NPME}{m}*100$ *NPME*: Number of medical devices pertinent to COVID-19-related requirements *m*: Total number of medical devices
Medical equipment condition (SH8)	% of flawed medical equipment	$\frac{NFME}{m}*100$ *NFME*: Number of flawed medical equipment *m*: Total number of medical devices
Presence of medical care procedures (SH9)	Design of medical care procedures related to COVID-19 management	If designed (1), otherwise (0)
Diffusion of procedures and protocols (SH10)	% of widespread COVID-19-related procedures and protocols	$\frac{NWPP}{p}*100$ *NWPP*: Number of widespread COVID-19-related procedures and protocols *p*: Total number of procedures and protocols
Compliance with medical care protocols and procedures (SH11)	Percentage of monitored adverse events in the ED	$\frac{NSAE}{ae}*100$ *NSAE*: Number of supervised adverse events related to COVID-19 patients *ae*: Total number of adverse events
Efficacy of radiology process (SH12)	Average turnaround time for radiology outcomes	$\overset{n}{\underset{i=1}{\sum }}\frac{DD_{i}-OD_{i}}{ANRT}$ *ANRT*: Annual number of radiology tests. *DD_i_*: Delivery date of radiology test i *OD_i_*: Order date of radiology test i
Efficacy of clinical lab (SH13)	Average turnaround time for lab test results	$\overset{n}{\underset{j=1}{\sum }}\frac{DD_{j}-OD_{j}}{ANLT}$ *ANLT*: Annual number of lab tests. *DD_j_*: Delivery date of lab test j *OD_j_*: Order date of laboratory test j
Efficacy of hospitalization process (SH14)	Average patient transfer time from the ED to hospitalization bed	$\overset{n}{\underset{k=1}{\sum }}\frac{\mathrm{RT}D_{k}-\mathrm{ST}D_{k}}{ANTP}$ *ANTP*: Annual number of transferred COVID-19 patients *RTD_k_*: Real transfer date for COVID-19 patient k *STD_k_*: Scheduled transfer date for COVID-19 patient k
Efficacy of pharmaceutical service (SH15)	Average lead time for medication delivery	$\overset{n}{\underset{l=1}{\sum }}\frac{DD_{l}-RD_{l}}{ANMO}$ *ANMO*: Annual number of medication orders *DD_l_*: Delivery date of medication order l *RD_l_*: Request date of medication order l
Transportation efficacy (SH16)	Availability of ambulances satisfying the WHO COVID-19 management standards	If available (1), otherwise (0)
Efficacy of sterilization process (SH17)	Implementation of sterilization protocols against COVID-19	If implemented (1), otherwise (0)
Efficacy of non-core activities (SH18)	Number of functioning non-core activities	Number of noncore activities that are currently underpinning ED operations
Number of specialists (SH19)	Amount of available positions for ED specialists	Amount of specialists necessitated in the ED for balancing the COVID-19 demand
Number of general practitioners (SH20)	Amount of available positions for ED general physicians	Amount of general physicians in the ED for balancing the COVID-19 demand
Certification in Advanced Life Support (SH21)	Percentage of medical staff with ALS certification	$\frac{NCMS}{NMS}*100$ *NCMS*: Number of certified medical staff *NMS*: Number of medical staff
Number of nurses (SH22)	Amount of available positions for ED nurses	Amount of nurses necessitated in the ED for balancing the COVID-19 demand.
Readiness of accessories and instrumentation (SH23)	Availability of accessories and instrumentation required by the ED for COVID-19 management	Number of medical accessories and instruments necessitated for balancing the COVID-19 demand
Supplies fill rate (SH24)	Inventory service level (medical consumables)	$\frac{ASOC}{o}*100$ *ASOC*: Amount of satisfied orders of medical consumables *o*: Total number of orders
Medication fill rate (SH25)	Inventory service level (medication)	$\frac{ASMO}{mo}*100$ *ASMO*: Amount of satisfied medication orders *mo*: Total number of medication orders
Bed occupancy rate (SH26)	Bed occupation ratio	$\frac{NBOcovid-19}{NBcovid-19}*100$ *NBOcovid*-19: Number of ED beds occupied by COVID-19 patients *NBcovid*-19: Total number of ED beds assigned to COVID-19 patients
Mean physician waiting time (SH27)	Average doctor’s waiting time	$\overset{n}{\underset{k=1}{\sum }}\frac{AT_{k}-CT_{k}}{ANCP}$ *ANCP*: Annual number of COVID-19 patients *AT_k_*: Arrival time for COVID-19 patient k *CT_k_*: Consultation time for COVID-19 patient k
Patient satisfaction (SH28)	Patient satisfaction ratio	$\frac{NSCP}{NCP}*100$ *NSCP*: Number of satisfied COVID-19 patients *NCP*: Number of COVID-19 patients admitted in the ED
Mean length of stay (SH29)	Mean length of stay in the ED	$\frac{TLS}{NCP}*100$ *TLS*: Total length of stay in the ED *NCP*: Number of COVID-19 patients admitted in the ED
Re-entry rate (SH30)	72-h readmission rate	$\frac{NRcovid-19}{NCP}*100$ *NRPT*: Number of readmitted COVID-19 patients within a 72-h period due to this disease *NCP*: Number of COVID-19 patients admitted in the ED
Waiting time for triage categorization (SH31)	Mean waiting time for triage categorization	$\overset{n}{\underset{k=1}{\sum }}\frac{AT_{k}-TCT_{k}}{NCP}$ *NCP*: Number of COVID-19 patients admitted in the ED *AT_k_*: Arrival time for COVID-19 patient k *TCT_k_*: Triage categorization time for COVID-19 patient k
Hospital-obtained infections (SH32)	Average monthly number of intra-hospital COVID-19 infection	$\frac{ANIHCI}{12}$ *ANIHCI*: Annual number of intra-hospital COVID-19 infections
Medication mistakes (SH33)	Average monthly number of medication mistakes	$\frac{ANMM}{12}$ *ANMM*: Annual number of medication mistakes
Clinical diagnosis mistakes (SH34)	Average monthly number of COVID-19 diagnosis mistakes	$\frac{ANCDM}{12}$ *ANCDM*: Annual number of COVID-19 diagnosis mistakes
Patient identification errors (SH35)	Average monthly number of patient misidentification mistakes	$\frac{ANPMM}{12}$ *ANPMM*: Annual number of patient misidentification mistakes

**Table 13:** Initial CoCoSo decision matrix *M*.

AW	D1	D2	D3	Weight
SH1	80	100	90	0.028
SH2	0	0	0	0.029
SH3	1	1	1	0.032
SH4	1	1	1	0.026
SH5	2200	2800	3000	0.029
SH6	100	100	95	0.038
SH7	98	95	95	0.039
SH8	0	0.5	1	0.043
SH9	1	1	1	0.039
SH10	99	100	95	0.039
SH11	0	0	0	0.039
SH12	90	60	120	0.019
SH13	90	60	60	0.020
SH14	15	15	20	0.016
SH15	5	60	5	0.019
SH16	1	1	1	0.019
SH17	1	1	1	0.019
SH18	0	0	0	0.022
SH19	6	3	8	0.029
SH20	30	4	36	0.035
SH21	60	80	90	0.037
SH22	26	23	20	0.036
SH23	68	85	100	0.025
SH24	95	95	90	0.028
SH25	90	95	90	0.025
SH26	40	90	70	0.031
SH27	5	5	7	0.024
SH28	85	91	90	0.026
SH29	60	60	65	0.025
SH30	0.1	1	0.5	0.025
SH31	5	5	5	0.024
SH32	0	0	0	0.028
SH33	0	0	0	0.029
SH34	0	0	0	0.032
SH35	0	0.002	0.001	0.030

**Table 14:** The sum of weighted comparability Si, power-weighted comparability sequences Pi, and aggregated appraisal scores (emergency department performance index—EDPI).

Emergency Department	*S*i	*P*i	*M*ia	*M*ib	*M*ic	*M*i
D1	0.74	27.89	0.350	2.605	0.933	2.243
D2	0.78	29.91	0.375	2.783	1.000	2.401
D3	0.55	21.91	0.275	2.000	0.732	1.740

## Data Availability

The data presented in this study are available within the Results section.
